# Selective haematological cancer eradication with preserved haematopoiesis

**DOI:** 10.1038/s41586-024-07456-3

**Published:** 2024-05-22

**Authors:** Simon Garaudé, Romina Marone, Rosalba Lepore, Anna Devaux, Astrid Beerlage, Denis Seyres, Alessandro Dell’ Aglio, Darius Juskevicius, Jessica Zuin, Thomas Burgold, Sisi Wang, Varun Katta, Garret Manquen, Yichao Li, Clément Larrue, Anna Camus, Izabela Durzynska, Lisa C. Wellinger, Ian Kirby, Patrick H. Van Berkel, Christian Kunz, Jérôme Tamburini, Francesco Bertoni, Corinne C. Widmer, Shengdar Q. Tsai, Federico Simonetta, Stefanie Urlinger, Lukas T. Jeker

**Affiliations:** 1 Department of Biomedicine, Basel University Hospital and University of Basel, Basel, Switzerland; 2 Transplantation Immunology & Nephrology, Basel University Hospital, Basel, Switzerland; 3 Cimeio Therapeutics, Basel, Switzerland; 4 Department of Hematology, Basel University Hospital, Basel, Switzerland; 5 Department of Laboratory Medicine, Diagnostic Hematology, Basel University Hospital, Basel, Switzerland; 6 Division of Hematology, Department of Oncology, Geneva University Hospitals, Geneva, Switzerland; 7 Department of Hematology, St. Jude Children’s Research Hospital, Memphis, TN USA; 8 Translational Research Center for Oncohematology, Department of Medicine, Faculty of Medicine, University of Geneva, Geneva, Switzerland; 9 Ridgeline Discovery, Basel, Switzerland; 10 ADC Therapeutics (UK), London, UK; 11 Institute of Oncology Research, Faculty of Biomedical Sciences, USI, Bellinzona, Switzerland; 12 Oncology Institute of Southern Switzerland, Ente Ospedaliero Cantonale, Bellinzona, Switzerland; 13 Innovation Focus Cell Therapy, Basel University Hospital, Basel, Switzerland; 14 Present Address: Centre de Recherches en Cancérologie de Toulouse, Université de Toulouse, Inserm, CNRS, Toulouse, France

**Keywords:** Cancer immunotherapy, Protein design, Genetic engineering, Leukaemia, Synthetic biology

## Abstract

Haematopoietic stem cell (HSC) transplantation (HSCT) is the only curative treatment for a broad range of haematological malignancies, but the standard of care relies on untargeted chemotherapies and limited possibilities to treat malignant cells after HSCT without affecting the transplanted healthy cells^1^. Antigen-specific cell-depleting therapies hold the promise of much more targeted elimination of diseased cells, as witnessed in the past decade by the revolution of clinical practice for B cell malignancies^2^. However, target selection is complex and limited to antigens expressed on subsets of haematopoietic cells, resulting in a fragmented therapy landscape with high development costs^2–5^. Here we demonstrate that an antibody–drug conjugate (ADC) targeting the pan-haematopoietic marker CD45 enables the antigen-specific depletion of the entire haematopoietic system, including HSCs. Pairing this ADC with the transplantation of human HSCs engineered to be shielded from the CD45-targeting ADC enables the selective eradication of leukaemic cells with preserved haematopoiesis. The combination of CD45-targeting ADCs and engineered HSCs creates an almost universal strategy to replace a diseased haematopoietic system, irrespective of disease aetiology or originating cell type. We propose that this approach could have broad implications beyond haematological malignancies.

## Abstract (web summary)

An antibody–drug conjugate that targets the pan-haematopoietic marker CD45 combined with transplanted stem cells engineered to be shielded from it can eradicate leukaemic cells while preserving haematopoiesis.

## Main

The finding that HSCs are transplantable and can reconstitute the entire haematopoietic system led to the dream of gently replacing a diseased haematopoietic system^1,6^. Although HSCT has become established clinical care, the development of highly effective antigen-specific, cell-depleting drug modalities, such as ADCs or chimeric antigen receptor (CAR) T cells, holds the promise of depleting HSCs and tumour cells alike in a targeted manner and could therefore profoundly improve HSCT^2,7–10^. However, shared antigen expression on tumour cells and essential healthy cells (such as HSCs or T cells) creates the risk of toxicity^3,11^. Moreover, the diversity of haematopoietic cell types and the complex, heterogeneous expression of hundreds of different surface antigens by haematological cancer cells make target selection difficult. Candidate antigens with a profile as favourable as CD19 are mostly elusive^3,4^. Given these limitations, we reversed the criteria for target selection: rather than emphasizing cell-type specificity of antigen expression, we hypothesized that an attractive target would be broadly expressed by all haematopoietic cells, including haematopoietic stem and progenitor cells (HSPCs), leukaemic stem cells and differentiated cells, but not non-haematopoietic cells. The receptor tyrosine phosphatase CD45 is expressed only by nucleated cells of haematopoietic origin and therefore represents a pan-haematopoietic marker. Furthermore, ADCs that target mouse CD45 effectively deplete long-term reconstituting HSCs (LT-HSCs), enabling syngeneic HSCT^12^. However, because CD45^+^ cells are essential for haematopoiesis, immune defence and many non-immune functions^13^, a highly effective CD45-targeting therapy could not be tolerated in the long term without extra precautions. Deleting CD45 on HSPCs, as has been proposed for non-essential genes such as *CD33* (refs. ^14–16^ and NCT04849910), is impossible because CD45 deficiency results in severe combined immunodeficiency^17^. However, we previously demonstrated that substituting a single amino acid in mouse CD45 abolished the binding of a monoclonal antibody but preserved CD45 expression and probably its function^18^. Various groups, including ours, have since engineered human HSPCs that were shielded from antigen-specific therapies but preserved the function and maintained engraftment and multilineage differentiation potential^19–21^. Therefore, we aimed to engineer CD45 to be resistant to a CD45-targeting ADC but to maintain the functionality of HSCs and thereby to enable targeted post-HSCT therapy. Here, we have developed a highly potent CD45-targeting ADC and identified a combination of base editor (BE) and guide RNA that can install a molecular shield in HSPCs so they retain long-term reconstitution potential in vivo. Notably, we have demonstrated complete and selective depletion of multiple haematological cancer cells in vivo without affecting base-edited HSCs and haematopoietic cells.

## CD45 regions for epitope engineering

**Fig. 1:**
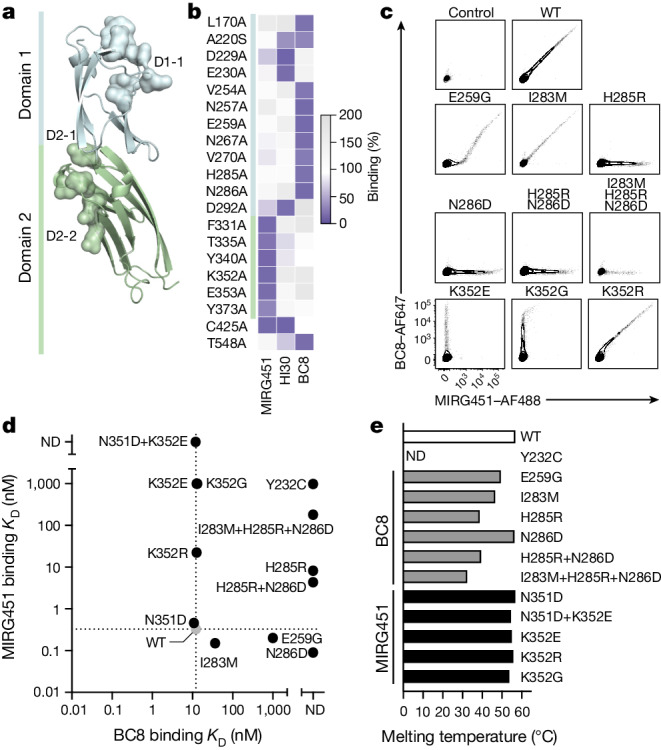
Identification of base-editable CD45 variants with stable biophysical properties. **a**, Crystal structure (retrieved from PDB under accession number 5FMV) of the CD45 domain 1 (blue) and domain 2 (green) highlighting selected regions D1-1, D2-1 and D2-2 as surface density. **b**, Heatmap showing CD45 alanine substitutions that result in less than 20% residual binding for one monoclonal antibody but have more than 70% for either of the other two tested anti-CD45 antibodies. **c**, Flow cytometry of CD45 variants overexpressed in DF-1 cells affecting the MIRG451 and BC8 epitopes (*n* = 4 biological replicates). WT, wild type. **d**, MIRG451 and BC8 binding affinity for purified recombinant CD45 D1–D2 protein variants measured by BLI. The grey circle refers to the wild type. *K*_D_, dissociation constant; ND, not detected. **e**, Melting temperature of purified recombinant CD45 D1–D2 protein variants (*n* = 1–2). Source Data

**Extended Data Fig. 1:**
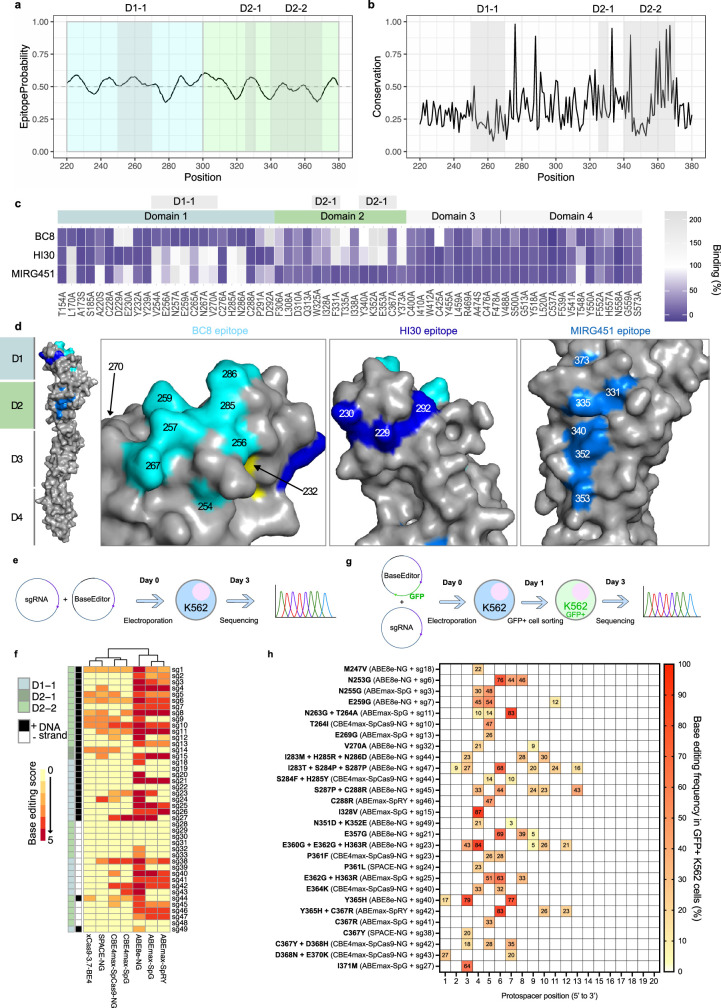
Identification of base editable CD45 variants. **a**, Epitope probability of each CD45 residue across domains 1 (blue) and 2 (green). **b**, Per-residue evolutionary conservation grade across domains 1 and 2 of CD45. **c**, Heatmap showing CD45 alanine substitutions resulting in a < 20% residual binding for one monoclonal antibody while maintaining >70% for other anti-CD45 tested antibodies. **d**, Human CD45 crystal structure containing domains 1-4 (PDB: 5FMV) is shown in grey. Key residues (<20% binding in Ala scan and >70% binding to other tested antibodies) of each epitope are highlighted in colour. Cyan for BC8, dark blue for HI30 and blue for MIRG451. From left to right, closer view of epitopes of BC8 in domain 1, HI30 epitope in domain 1 and MIRG451 in domain 2. Y232A (in yellow) reduced binding to all three antibodies and is not fully surface exposed. L170 and A220 (belonging to the BC8 epitope) are not depicted, since CD45 D1-4 (residues 223-571 only) was used for the crystallization. **e**, Base editor screening experimental timeline. Plasmids encoding sgRNAs and base editors were co-electroporated in K562 cells and bulk cells were sequenced 3 days later (Sanger). **f**, Heatmap showing editing rates of different base editors screened against designed sgRNAs. Each sgRNA is colour-coded with its CD45 sub-domain and targeted DNA strand. Base efficiencies are represented by a custom base editor score. **g**, Base editor validation screening experimental timeline. Plasmids encoding sgRNAs and base editors (GFP cassette within base editor plasmids) were co-electroporated in K562 cells and GFP+ cells were sorted and sequenced 3 days later (Sanger). **h**, Heatmap displaying the base editing efficiency per protospacer position in GFP + K562 cells for each unique editing profile determined from (**e**).

We computationally examined the CD45 extracellular domain (ECD) to identify regions suitable for epitope engineering. To identify broadly applicable cell-shielding variants, we focused on non-conserved ECD regions. Domains 1 and 2 (D1 and D2) display unique topologies with structural variations from the typical fibronectin type III β-sandwich, whereas D3 and D4 have the classical fibronectin type III fold^22^. We therefore prioritized the distinctive D1 and D2 domains (Fig. 1a) and avoided residues that are known or predicted to undergo post-translational modifications (such as glycosylation), as well as those involved in the stabilization of domain–domain interfaces, through hydrophobic interactions and salt bridges^22^. Furthermore, we assessed surface accessibility, epitope probability and per-residue evolutionary conservation across D1 and D2. These criteria indicated that regions D1-1, D2-1 and D2-2 were ideal candidates to identify protein sites able to tolerate mutations without compromising the structural or functional integrity of CD45 (Fig. 1a and Extended Data Fig. 1a,b). Next, alanine (Ala) scanning of the entire CD45 ECD identified specific residues that affected the binding of three different mAbs (BC8, HI30 and MIRG451). BC8 is the parental mAb of the clinically most-advanced antibody targeting CD45 and binds to D1 (NCT02665065), whereas HI30 and MIRG451 bind to D1 and D2, respectively. Preliminary epitope residues were defined as having mAb binding of up to 20% to the alanine variants, compared with wild-type CD45 (CD45^wt^), for one mAb and more than 70% for either of the other two mAbs that served as controls (Fig. 1b). Candidate residues were mapped to the CD45 crystal structure^22^ (PDB: 5FMV) and analysed for their structural localization, spatial proximity and solvent exposure. BC8 and HI30 showed two clusters of proximal epitope residues, partly overlapping at the N-terminal part of D1, whereas the MIRG451 epitope residues were located in D2, facing the opposite side of the ECD surface (Fig. 1b and Extended Data Fig. 1c,d). Substitutions to alanine that affected all the antibodies were excluded because they may indirectly affect antibody binding, for example through altered structure, conformation or expression of the alanine variants (Extended Data Fig. 1c). The structural localization of the remaining sites was analysed to distinguish true epitope sites from indirect ones. For instance, T548 (D4) is far away from the mapped epitope residues (D1 and D2) and therefore cannot directly affect BC8 or HI30 binding (Fig. 1b). Furthermore, Y232 is buried in the protein with only 4% surface accessibility (Extended Data Fig. 1d). We conclude that these residues represent false positives that are not part of the actual epitopes. Notably, Y232 had been identified as a key structural residue that was thought to have a role in positioning the mucin-like extension at the N terminus of the ECD^22^. Mutating it could therefore affect protein structure. The effects of alanine mutations on cysteine residues were not considered, owing to the importance of disulfide bridges in the ECD structure. In summary, these findings validated our initial computational predictions and rationale for prioritizing domains D1 and D2, and also emphasized the importance of complementary assessments for accurate identification of the epitope landscape.

Next, we assessed the feasibility of using BEs to generate antibody-discernible amino acid substitutions in D1-1, D2-1 and D2-2. BEs constitute a class of genome editors that is based on a catalytically impaired CRISPR–Cas nuclease fused to a deaminase. Cytosine BEs (CBEs) catalyse C to T, whereas adenine BEs (ABEs) catalyse A-to-G conversions in an editing window of several nucleotides, generating rather circumscribed genomic base conversions with high editing efficiencies^23^. We screened multiple BEs representing different architectures with all single guide RNAs (sgRNAs) compatible with an NG(N) protospacer adjacent motif (PAM) on both DNA strands and a predicted BE window targeting nucleotides in regions of interest (Extended Data Fig. 1e–h, Supplementary Tables 1 and 2 and Supplementary Text 1). Taking into account editing efficiencies, precision (that is, bystander editing) and the physicochemical and structural properties of the inferred amino acid substitutions, we selected CD45^E259^ and CD45^N286^ (both BC8) and CD45^K352^ (MIRG451) for subsequent experiments.

## Characterization of CD45 variants

**Extended Data Fig. 2:**
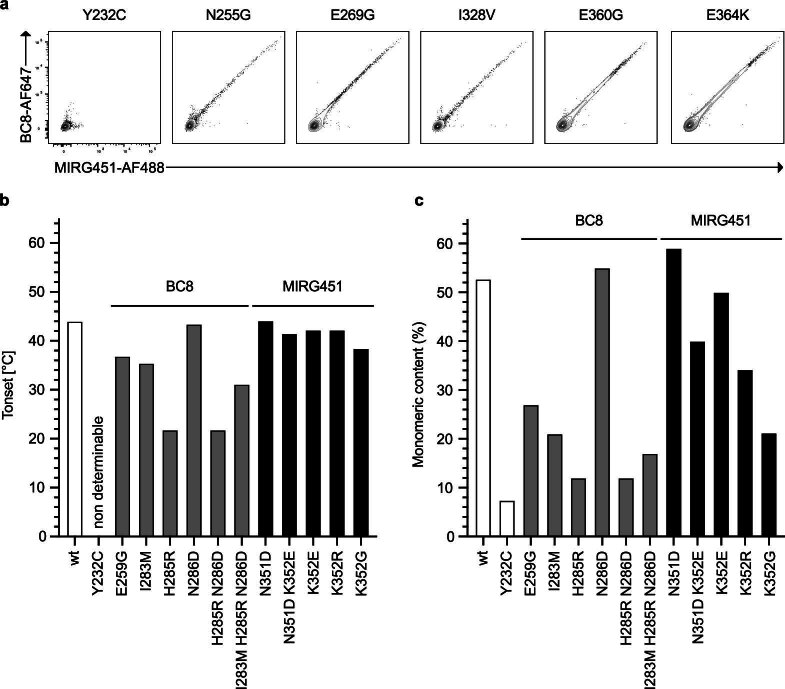
Biophysical property assessment of base editable CD45 variants. **a**, Flow cytometry of CD45 variants overexpressed in DF-1 cells affecting MIRG451 and BC8 epitopes (n = 4 biological replicates, n = 2 for Y232C). On-set temperatures (**b**) and monomeric content (**c**) of purified recombinant CD45 D1-D2 protein variants (n = 1-2).

To characterize candidate CD45 shielding variants, we overexpressed individual substitutions or combinations of them (resulting from bystander editing) and used flow cytometry to measure binding to BC8 and MIRG451. Compared with CD45^WT^, CD45^E259G^ showed reduced binding to BC8 but intact binding to MIRG451 in CD45^low^ cells, whereas CD45^high^ cells demonstrated residual binding to BC8, a pattern we previously classified as intermediate (low) binders^19^ (Fig. 1c). Other substitutions affected neither expression nor binding to either mAb, and CD45^Y232C^ abolished binding of both mAbs (Extended Data Fig. 2a). Next, we investigated CD45^N286D^ and the bystander triple mutant CD45^I283M+H285R+N286D^ generated by ABE8e–NG plus sgRNA-44, as well as its potential single and double substitutions (Extended Data Fig. 1h). CD45^I283M^ did not alter mAb binding by itself. By contrast, CD45^H285R^ and CD45^N286D^ completely abolished BC8 binding when overexpressed individually or combined, but MIRG451 binding remained intact. However, the triple mutant resulted in reduced MIRG451 binding, which may indicate impaired protein stability and/or folding (Fig. 1c). These results confirmed that residues E259, H285 and N286 are important for BC8 binding to CD45 D1. Next, we examined the MIRG451 epitope on CD45 D2. ABE8e–NG plus sgRNA-49 resulted in CD45^N351D+K352E^ affecting a key residue of the MIRG451 epitope (K352) (Fig. 1b and Extended Data Fig. 1c,d,g,h). However, because lysines (K) are encoded by AAA or AAG codons, more substitutions could arise from ABE, that is CD45^K352E^ (GAG/GAA), CD45^K352G^ (GGG/GGA) or CD45^K352R^ (AGG/AGA). CD45^K352E^ and CD45^K352G^ individually abolished the binding of MIRG451 but did not impair BC8 binding, whereas CD45^K352R^ barely affected MIRG451 binding without altering BC8 (Fig. 1c). Thus, K352 constitutes a key residue of the MIRG451 epitope (CD45 D2), and CD45^K352E^ and CD45^K352G^ completely abolished MIRG451 binding.

To quantify shielding from antibody binding and determine the biophysical properties of the most promising protein variants, we produced human CD45 D1–D2 recombinantly and assessed BC8 and MIRG451 binding affinities by biolayer interferometry (BLI). Affinities for CD45^WT^ were 10–20 nM (BC8) and less than 1 nM (MIRG451), respectively (Fig. 1d). BLI measurements confirmed the overexpression results (Fig. 1c,d): full BC8 shielding (CD45^H285R^, CD45^N286D^, CD45^H285R+N286D^ and CD45^I283M+H285R+N286D^), partial BC8 shielding (CD45^E259G^) and CD45^WT^-like binding were observed. However, all variants containing CD45^H285R^ also affected binding of MIRG451, a mAb binding a distant epitope. As outlined above, we had concluded that CD45^Y232^ was not part of the BC8 epitope. Nevertheless, we included CD45^Y232C^ in the BLI analysis because it has been reported to shield from a CAR T cell targeting CD45 when engineered by BE^21^. Recombinant CD45^Y232C^ was difficult to produce, but the small fraction of purified monomeric protein confirmed that there was no BC8 or MIRG451 binding (Fig. 1d). By contrast, all variants with K352 mutations successfully retained BC8 binding and displayed a large loss in MIRG451 binding, as intended. We also characterized the thermal stability of CD45 variants by measuring the temperatures at which the protein begins to unfold (*T*_onset_) and at which 50% of the protein is unfolded (*T*_M_). Furthermore, we determined the aggregation propensity (monomeric content), because our antibody binding results indicated that multiple CD45 variants altered the protein structure across multiple domains. CD45^E259G^, CD45^I283M^, CD45^H285R^, CD45^H285R+N286D^ and CD45^I283M+H285R+N286D^ displayed decreased thermal stability, with some being partly denatured at physiological temperatures (Fig. 1e and Extended Data Fig. 2b). CD45^Y232C^ exhibited a high fluorescence background, which is probably the result of improper folding, aggregation or partial denaturation of this variant at 25 °C. Consequently, no thermal denaturation was observed. By contrast, CD45^N286D^, CD45^N351D^, CD45^N351D+K352E^ and all single CD45^K352^ variants had CD45^WT^-like thermal stabilities (Fig. 1e and Extended Data Fig. 2b), indicating that the substitutions had little or no effect on protein integrity. Furthermore, the monomer content of recombinant CD45^N286D^, CD45^N351D^ and CD45^K352E^ was almost identical to that of CD45^WT^. CD45^N351D+K352E^ and CD45^K352R^ showed a small reduction in monomeric content, but all other variants reduced the monomeric content of CD45 to a greater extent (Extended Data Fig. 2c). Thus, CD45^N286D^ and CD45^K352E^ displayed the most favourable biophysical characteristics.

## Optimized BE to shield HSPCs

**Fig. 2:**
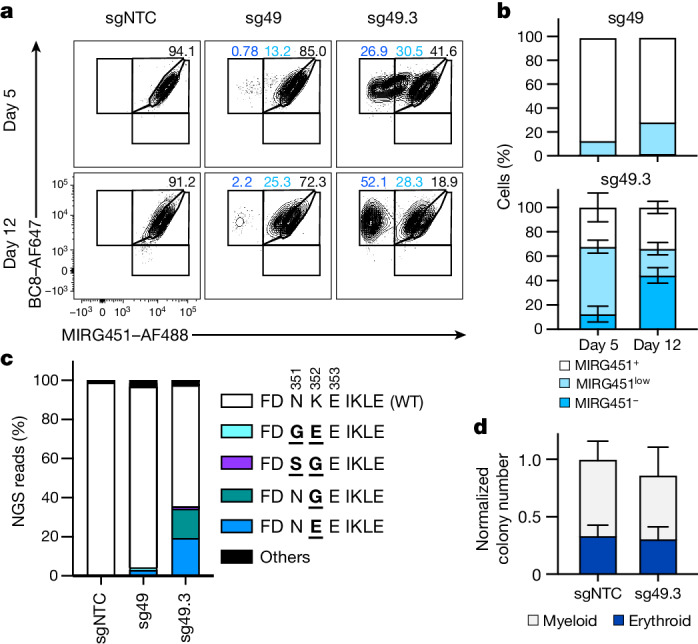
Optimized BE enables efficient ex vivo shielding of HSPCs. **a**, Flow cytometry of human CD34^+^ HSPCs 5 and 12 days after electroporation with ABE8e–SpRY mRNA and different sgRNAs. Black, MIRG451^+^ gate; light blue, MIRG451^low^ gate; dark blue, MIRG451^–^ gate. **b**, Quantification of cell subpopulations (from **a**) 5 and 12 days after electroporation with ABE8e–SpRY mRNA and sgRNA-49 (sg49; *n* = 2 biological replicates) or sgRNA-49.3 (sg49.3; *n* = 3). **c**, Results of NGS showing amino acid substitution profiles from sgRNA-NTC (sgNTC), sg49 and sg49.3 5 days after electroporation (*n* = 1). NGS reads accounting for less than 0.8% of total reads were classified as ‘others’. **d**, Quantification of myeloid and erythroid colonies formed in colony-forming units (CFUs) assay using HSPCs with ABE8e–SpRY mRNA and sg49.3 (*n* = 3 with two technical replicates each). Data are presented as mean ± s.d. Source Data

**Extended Data Fig. 3:**
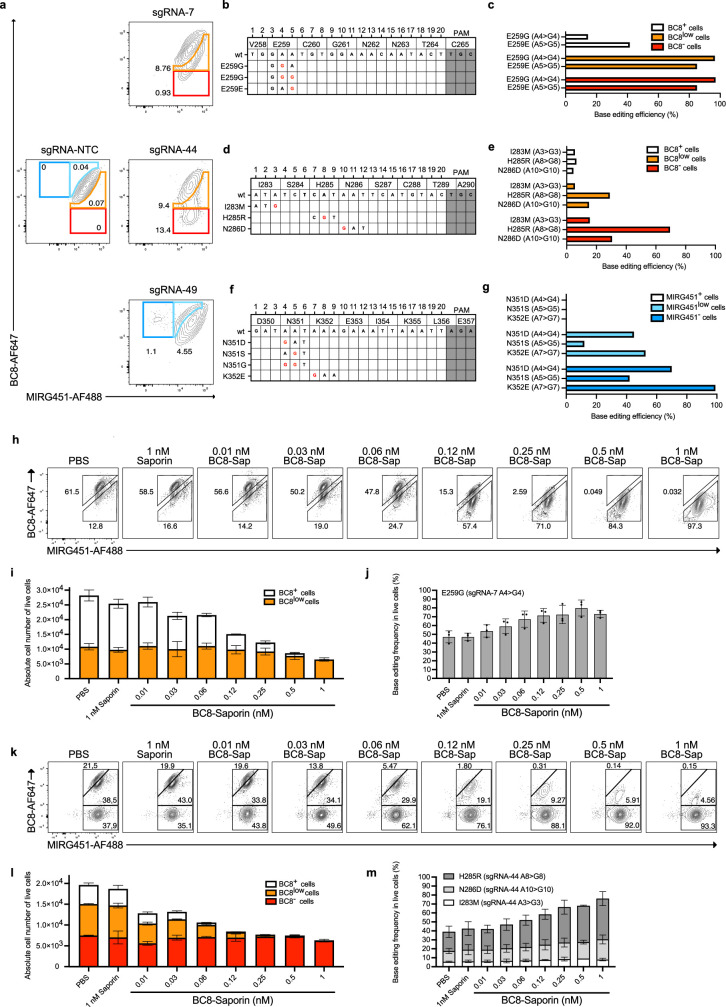
Base editing shields primary cells from a CD45 surrogate ADC. **a**, Flow cytometry of human activated T cells 5 days after electroporation of ABE8e-NG mRNA with sgRNA-NTC, sgRNA-7, sgRNA-44 or sgRNA-49. **b**, sgRNA-7 mapped on corresponding gDNA codons **c**, Bar graphs of Sanger sequencing results showing amino acid substitutions in sorted populations from sgRNA-7 edited cells (matching colour code). **d**, sgRNA-44 mapped on corresponding gDNA codons **e**, Bar graphs of Sanger sequencing results showing amino acid substitutions in sorted populations from sgRNA-44 edited cells (matching colour code). **f**, sgRNA-49 mapped on corresponding gDNA codons **g**, Bar graphs of Sanger sequencing results showing amino acid substitutions in sorted populations from sgRNA-49 edited cells (matching colour code) (In (**c,e,g**), n = 2 biological replicates). **h**, Flow cytometry panels of base edited human activated T cells (ABE8e-NG + sgRNA-7) incubated with increasing concentration of BC8-Saporin for 3 days. **i**, Quantification of the absolute number of living cells post-killing from (**h**) and their BC8 phenotype. **j**, Bar graphs showing the percentage of E259G (sgRNA-7 A4 > G4) base conversion in live cells for each BC8-Saporin concentration. **k**, Flow cytometry panels of base edited human activated T cells (ABE8e-NG + sgRNA-44) incubated with increasing concentration of BC8-Saporin for 3 days. **l**, Quantification of the absolute number of living cells post-killing from (**k**) and their BC8 phenotype. **m**, Bar graphs showing the percentage of E259G (sgRNA-7 A4 > G4) base conversion in live cells for each BC8-Saporin concentration (In (**i,j,l,m**), n = 3 biological replicate containing each 3 technical replicates). Each bar graph from **c, e, g, i, j, l** and **m** is plotting mean values +/− SD of biological replicates.

**Extended Data Fig. 4:**
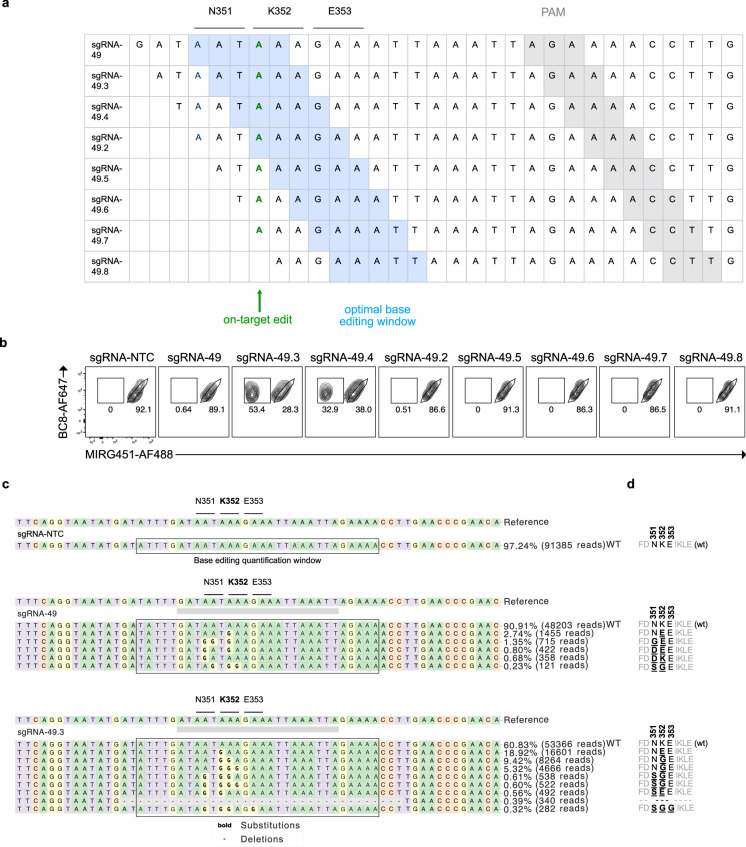
Optimization of base editing in HSPCs. **a**, Sequences of sgRNA-49 and of sgRNA-49.2 to 49.8 tiled along the K352 codon. The classical base editing window (position 4-8) is highlighted in blue and the intended A > G on-target is in bold green for each sgRNA. **b**, Flow cytometry of human CD34+ HSPCs base edited with ABE8e-SpRY mRNA and each sgRNA from (**a**) (12 days post-electroporation). **c**, Next generation sequencing of HSPCs 5 days after electroporation with ABE8e-SpRY mRNA and different sgRNAs. CRISPResso plots depict a 60 base pairs region, comprising 30 bases flanking upstream and downstream from the center of each sgRNA (aligned to sgRNA-49.3 for sgRNA-NTC). The quantification of base conversions is set on a 30 base pairs window, with 15 bases flanking upstream and downstream from the center of each sgRNA (aligned to sgRNA-49.3 for sgRNA-NTC). NGS reads accounting for less than 0.8% of total reads were classified as “Others”. **d**, Summary of amino acid conversions at positions N351, K352, and E353 across different sequencing profiles depicted in (**c**) for sgRNA-NTC, sgRNA-49, and sgRNA-49.3.

To investigate BE-mediated cell shielding in primary cells, we used human T cells electroporated with ABE8e–NG mRNA plus sgRNA and BC8–Saporin^12^ as a surrogate ADC (Extended Data Fig. 3 and Supplementary Text 2). Although we demonstrated feasibility with CD45^E259G^ and CD45^I283M+H285R+N286D^ (plus bystander edits), editing efficiency for the preferred variant, CD45^K352E^ (using ABE8e–NG plus sgRNA-49), was very low (Extended Data Fig. 3a). To increase CD45^K352E^ editing we repositioned ABE8e^24^, the most efficient BE from the screening (Extended Data Fig. 1f), using the almost-PAMless SpRY Cas9 (ref. ^25^) to tile seven sgRNA-49 daughter sgRNAs along the CD45^K352^ codon (Extended Data Fig. 4a and Supplementary Table 2). We electroporated mobilized CD34^+^ peripheral blood HSPCs with ABE8e–SpRY mRNA and sgRNA-49 or sgRNA-49.2 to sgRNA-49.8 and analysed the editing outcome by flow cytometry. Notably, sgRNA-49.3 and sgRNA-49.4 increased the MIRG451^−^ cell population from 0.6% to 53.4% and 32.9%, respectively (Extended Data Fig. 4b). Next-generation sequencing (NGS) confirmed strongly increased CD45^K352E^ and CD45^K352G^ with minor bystander editing (Extended Data Fig. 4c). Therefore, ABE8e–SpRY plus sgRNA-49.3 emerged as the most suitable BE combination. The shielding process was dynamic, resulting in a distinct population of around 50% shielded HSPCs by day 12 (Fig. 2a,b). NGS confirmed the dominant editing outcome of CD45^K352E^ and CD45^K352G^ with minimal CD45^N351S+K352G^ bystander editing with sgRNA-49.3 (Fig. 2c). We designated these HSPCs expressing two biophysically favourable shielding variants as ^sg49.3^HSPCs. They formed equal colony numbers, and the relative numbers of myeloid and erythroid colonies were similar to those of control HSPCs (designated ^sgNTC^HSPCs), indicating that they were functionally intact (Fig. 2d).

## Generating a potent humanized CD45-targeting ADC

**Extended Data Fig. 5:**
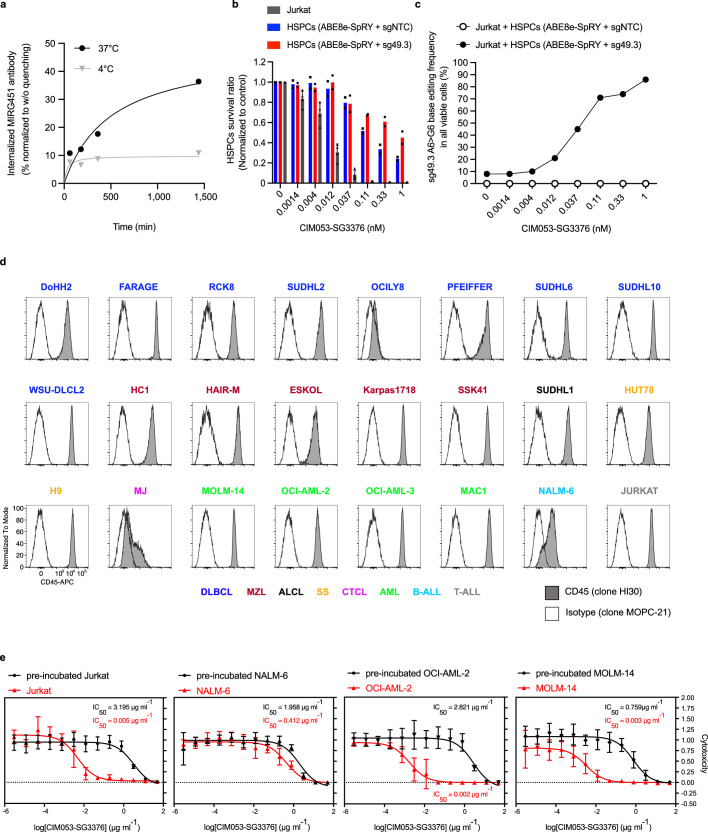
In vitro selective tumour killing and enrichment of engineered HSPCs. **a**, MIRG451 antibody internalisation percentage in Jurkat cells incubated at 37 °C and 4 °C (n = 1). **b**, Survival ratios of control (sgNTC) HSPCs, edited (sg49.3) HSPCs and Jurkat from HSPCs:Jurkat co-cultures for 72 h in the presence of CIM053-SG3376 at different concentrations. Data are normalised to untreated samples and each bar graph is plotting mean values +/− SD of 3 technical replicates. **c**, Frequency of sg49.3 A6 > G6 base conversion (K352E/G) in all sorted live cells from co-culture conditions 72 h after incubation with each CIM053-SG3376 concentration (n = 1). **d**, Flow cytometry panels showing CD45 expression in 24 leukaemia/lymphoma cell lines representing B, T and myeloid differentiation. **e**, In vitro killing curves of mCherry-luciferase labelled tumour cell lines (Jurkat, NALM-6, OCI-AML-2 and MOLM-14) pre-incubated or not with CIM053-SG3376 at different concentrations (n = 2 biological replicates with each 2-3 technical replicates, normalised to untreated conditions, data are presented as mean values +/− SD).

Next, we optimized the cell depleter for in vivo experiments. Around 40% of the MIRG451 mAb was internalized by Jurkat cells, which is a prerequisite for ADC-mediated cytotoxicity (Extended Data Fig. 5a). We therefore humanized and engineered MIRG451 (fragment crystallizable (Fc) region silent) for potential clinical development, resulting in CIM053, and stochastically conjugated it with the linker pyrrolobenzodiazepine (PBD) dimer payload SG3376 (ref. ^26^). The PBD dimer in SG3376 is very similar to the highly potent PBD dimer SG3199 used in tesirine, which is applied clinically^27^, except that it contains a non-cleavable linker to avoid the nonspecific bystander killing of shielded cells. We investigated whether CIM053–SG3376 could selectively kill tumour cells in vitro but spare co-cultured ^sg49.3^HSPCs. Increasing concentrations effectively killed Jurkat cells (a T-cell leukaemia cell line) and unedited HSPCs when co-cultured in vitro (Extended Data Fig. 5b). For comparison, ^sg49.3^HSPCs were more resistant to increasing ADC concentrations (Extended Data Fig. 5b). Some ^sg49.3^HSPC killing was expected, because about half of the cells remained unedited. As a consequence, increasing ADC concentrations strongly enriched for the A6 > G6 edited genotype among all surviving cells (Extended Data Fig. 5c). To investigate whether a CD45-targeting ADC could be used broadly, we analysed CD45 expression in 24 haematological cancer cell lines representing major cell lineages, including B, T and myeloid cells. All except three (OCILY8, MJ and NALM-6) strongly expressed CD45 (Extended Data Fig. 5d). Furthermore, mCherry–luciferase-marked tumour cell lines representing lymphoid (Jurkat (T) and NALM-6 (B)) and myeloid (OCI-AML-2 and MOLM-14, both of which represent acute myeloid leukaemia (AML)) lineages were effectively killed by CIM053–SG3376 in vitro (Extended Data Fig. 5e). Cytotoxicity must have been CD45 dependent, because pre-incubation with unconjugated CIM053 prevented cell killing. Notably, NALM-6 had low CD45 expression and required higher doses for cytotoxicity. Collectively, these results underlined that CD45 could be a broadly applicable target and that CIM053–SG3376 effectively depleted CD45-expressing cells in vitro.

## Shielded HSPCs retained function in vivo

**Fig. 3:**
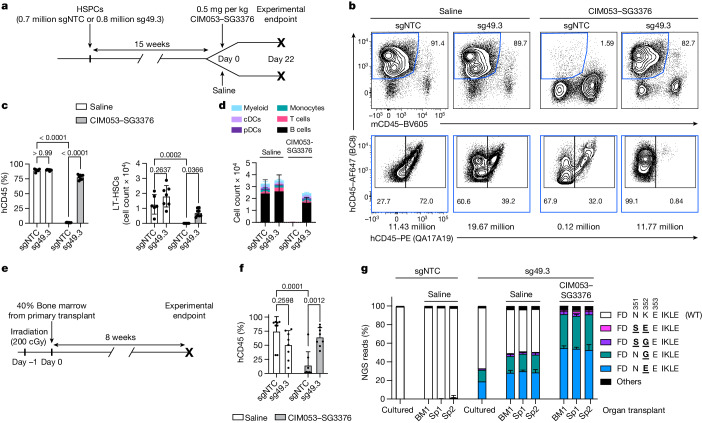
Engineered HSPCs retain function and are shielded from CIM053–SG3376 in vivo. **a**, Experimental timeline of NBSGW primary host mice (4 weeks old) humanized with control (sgNTC) or edited (sg49.3) HSPCs. Mice were subsequently treated with saline or CIM053–SG3376. **b**, Flow-cytometry analysis depicting the percentage of human chimerism (BC8^+^mCD45^−^) (top row, blue gate) in the BM of mice 22 days after treatment. Percentage of edited (BC8^+^QA17A19^−^) and unedited (BC8^+^QA17A19^+^) cells in the human cell population (bottom row). Absolute cell counts of representative panels, calculated according to input counting beads, are shown below each panel. **c**, Quantification of human chimerism (BC8^+^mCD45^−^) and LT-HSCs (BC8^+^mCD45^−^CD34^+^CD38^−^CD90^+^CD45RA^−^) in the BM of all mice. Selected *P* values are shown. **d**, Multi-lineage differentiation in the blood of all animals (conventional dendritic cells (cDCs); plasmacytoid dendritic cells (pDCs)). **e**, Experimental timeline of secondary transplant in NSG–SGM3 host mice (4 weeks old). **f**, Quantification of human chimerism (BC8^+^mCD45^−^) in the BM of secondary host mice. Selected *P* values are shown. **g**, NGS of matching electroporated cells cultured in vitro and organs from primary (bone marrow, BM; spleen, Sp1) and secondary (spleen, Sp2) host transplants. NGS reads accounting for less than 0.8% of total reads were classified as ‘others’ (*n* = 6–8 mice per group in **a–f**, 8 mice per group in **g**; data are mean ± s.d.). Ordinary two-way analysis of variance (ANOVA) tests (significance level, *α* = 0.05) were used to assess statistical difference between groups. Source Data

**Extended Data Fig. 6:**
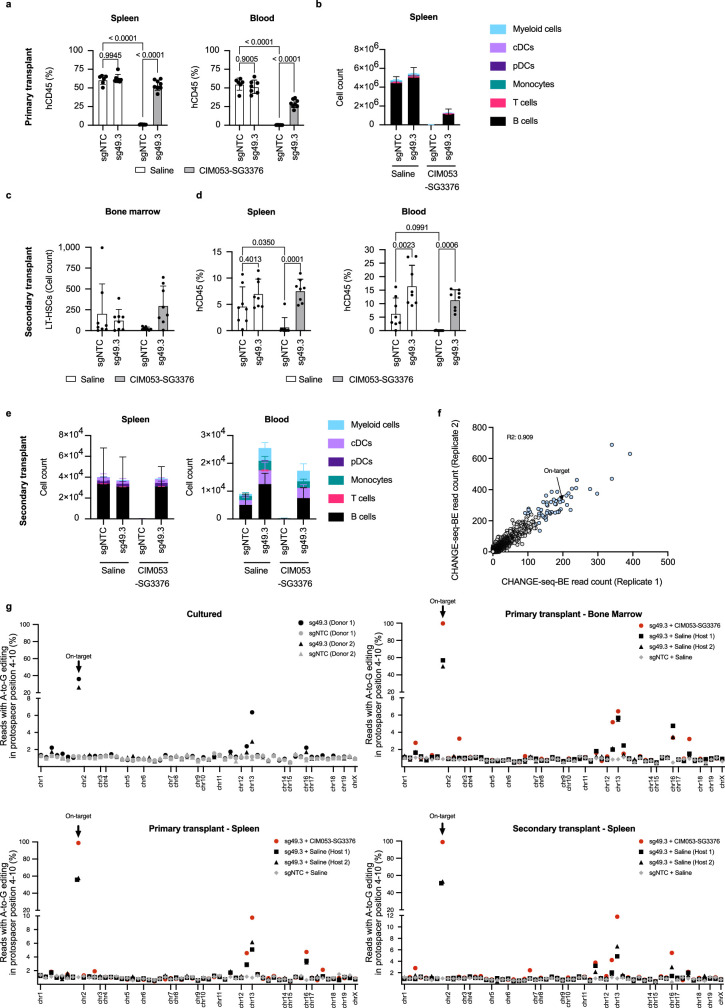
Engineered HSPCs retain function and are shielded from CIM053-SG3376 in vivo and display a good safety profile. **a**, Quantification of the human chimerism (%BC8^+^mCD45^−^) in the spleen and blood of all primary host mice. **b**, Multi-lineage differentiation in the spleen of all primary host mice. **c**, Quantification of long-term haematopoietic stem cells (LT-HSC) (%BC8^+^mCD45^−^CD34^+^CD38^−^CD90^+^CD45RA^−^) in the bone marrow of secondary host mice. **d**, Quantification of the human chimerism (%BC8^+^mCD45^−^) in the spleen and blood of all secondary host mice. **e**, Multi-lineage differentiation in the spleen and blood of all secondary host mice. (8 mice per group in all panels, plotting mean values +/− SD) (Ordinary two-way ANOVA tests (alpha 0.05) were employed to assess statistical difference between groups). **f**, Scatterplot representing co-relation between two technical replicates for CHANGE-seq-BE read counts. Top 50 potential off-target sites highlighted in blue were used for rhAmpSeq analysis and other possible off-targets are in plotted in grey. **g**, Scatterplots depicting A > G base editing percentages across positions 4 to 10 within the editing window for the top 50 rhAmpSeq sites. The plots are organized based on chromosomal coordinates and categorized by sample types, with cultured cells in the top-left, primary transplant bone marrow in the top-right, primary transplant spleen in the bottom-left, and secondary transplant spleen in the bottom-right (2 different donors for cultured cells, 2 hosts for sg49.3+Saline, 1 host for sgNTC+Saline and sg49.3 + CIM053-SG3376). Source Data

To investigate engraftment and differentiation potential, as well as in vivo shielding from ADC cytotoxicity, we injected ^sgNTC^HSPCs or ^sg49.3^HSPCs into NBSGW mice, which are immunodeficient and support engraftment and differentiation of human haematopoietic cells (Fig. 3a). Then, 15 weeks after HSPC transplantation, ^sgNTC^HSPC and ^sg49.3^HSPC groups were treated with either saline or CIM053–SG3376. Three weeks later, bone marrow (BM), spleen and blood were analysed. Human–mouse chimerism was indistinguishable between saline-injected mice engrafted with ^sgNTC^HSPCs or ^sg49.3^HSPCs (Fig. 3b,c and Extended Data Fig. 6a). Both groups showed nearly 90% human chimerism in BM and 50–60% in spleen and blood. A single dose of 0.5 mg per kg CIM053–SG3376 administered intravenously was well tolerated and completely depleted all human cells from all examined organs in mice engrafted with ^sgNTC^HSPCs (Fig. 3b,c and Extended Data Fig. 6a). The depletion must have been human CD45 (hCD45) specific because mouse CD45^+^ cells were relatively enriched (Fig. 3b, top). By contrast, mice engrafted with ^sg49.3^HSPCs maintained high human chimerism, showing that there was successful shielding (Fig. 3b,c and Extended Data Fig. 6a). All the remaining human cells in this group expressed hCD45 (BC8), but the antibody specific for the target epitope (QA17A19) could not bind, confirming that ^sg49.3^HSPCs and their progeny were completely shielded from CIM053–SG3376 (Fig. 3b, bottom). LT-HSCs were also completely depleted by CIM053–SG3376 in the unedited group but preserved in the shielded group (Fig. 3c). Together, these results demonstrate that there was normal engraftment and successful in vivo protection of the base-edited cells, despite the high potency of CIM053–SG3376. Analysis of cell types in blood and spleen demonstrated comparable multi-lineage cell-differentiation potential among the saline-treated ^sgNTC^HSPC and ^sg49.3^HSPC and the ADC-treated ^sg49.3^HSPC groups, and all cell types were depleted in the ADC-treated ^sgNTC^HSPC group (Fig. 3d and Extended Data Fig. 6b). To test the editing of HSCs with true long-term repopulation potential and preserved LT-HSC function, despite ADC exposure, 40% of the BM from each primary transplant recipient was injected into a secondary host mouse (Fig. 3e). Eight weeks after HSCT, secondary recipients showed engraftment in BM (Fig. 3f) and clearly detectable LT-HSCs (Extended Data Fig. 6c). Human cells were present in spleen and blood, and cell subsets also differentiated in secondary hosts (Extended Data Fig. 6d,e). Importantly, CIM053–SG3376 administration in the primary host did not affect the secondary engraftment of shielded ^sg49.3^HSPCs (Fig. 3f and Extended Data Fig. 6c). ADC-treated unedited cells remained very low (Fig. 3f) and did not give rise to cells in spleen and blood in most mice (Extended Data Fig. 6d,e). Furthermore, the edited cells remained QA17A19 negative, confirming the persistence of shielded HSPCs after serial transplants. The data were corroborated by NGS: around 50% of reads detected in cells isolated from the BM and spleen of primary recipients engrafted with ^sg49.3^HSPCs were edited and displayed a comparable ratio of CD45^K352E^ and CD45^K352G^ (Fig. 3g). Notably, relative editing frequencies remained constant in secondary hosts for a duration of 26 weeks in vivo. Application of CIM053–SG3376 eliminated all CD45^WT^ reads, resulting in complete enrichment of edited cells.

To nominate potential off-target editing sites we performed CHANGE-seq-BE^28^ on genomic DNA from the same donor used in this engraftment experiment (Extended Data Fig. 6f). The top 50 nominated off-target sites in each replicate, the on-target site and a perfect match off-target site were included for validation in HSPCs. All 58 nominated off-target sites were intergenic or intronic. We performed multiplexed targeted amplicon sequencing and analysis (rhAmpSeq) on ^sgNTC^HSPCs and ^sg49.3^HSPCs edited in vitro from this and a second donor, as well as cells isolated from the spleen and BM from primary and secondary host mice with or without ADC treatment. Considering a false discovery rate (FDR) of up to 0.05 and a difference in editing frequency of 1% or more, a total of nine off-target sites were validated in at least one sample with low editing frequencies (Extended Data Fig. 6g and Supplementary Table 3). However, these sites were not common in all samples; for example, in cultured HSPCs, only three (donor 1) and one (donor 2) off-target sites were validated. Cells isolated from mice did not show higher off-target editing frequencies, indicating that mutated off-target sites did not confer a selective advantage to the injected cells. Notably, CIM053–SG3376 treatment enriched edited cells, and thus the editing rate at these nine off-target sites was slightly increased (Extended Data Fig. 6g). Collectively, these results confirm that ^sg49.3^HSPCs allowed normal HSC engraftment and preserved multi-lineage differentiation without altered competitive fitness. Off-target analysis suggested a reasonable safety profile that could be improved further with high-fidelity engineered BEs, BEs with a more restrictive PAM or RNA–DNA hybrid guide RNAs^29,30^. Furthermore, the new CD45-targeting ADC, CIM053–SG3376, effectively depleted haematopoietic cells, including LT-HSCs, in vivo, but ^sg49.3^HSPCs and their progeny were successfully shielded from in vivo ADC killing.

## Precise haematological cancer depletion

**Fig. 4:**
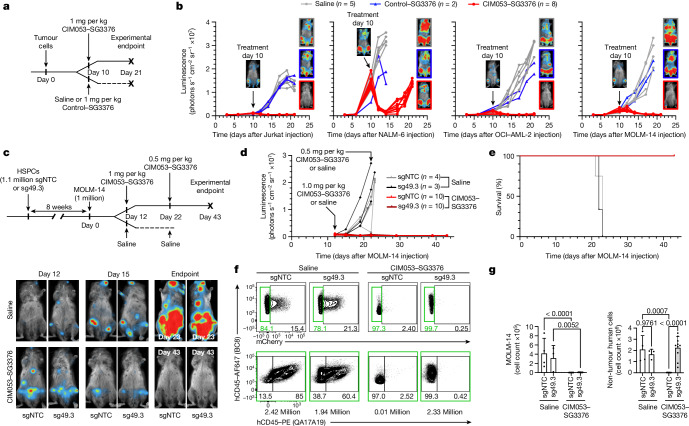
In vivo CIM053–SG3376-mediated selective tumour eradication with preserved haematopoiesis in edited cells. **a**, Experimental timeline of tumour xenografts in NBSGW host mice (5–6 weeks old) treated with saline, control SG3376 or CIM053–SG3376. **b**, Luminescence curves and images depicting representative mice from each treatment arm before treatment (day 10 after tumour injection) and on the day they were euthanized (*n* = 5 mice for saline, *n* = 2 for control SG3376 and *n* = 5 for CIM053–SG3376). **c**, Experimental timeline of NBSGW mice (4 weeks old) humanized with control (sgNTC) or edited (sg49.3) HSPCs, followed by MOLM-14 tumour xenograft injection. The mice were subsequently treated with either saline or CIM053–SG3376. Luminescence images of representative mice were taken before treatment (day 12 after MOLM-14 injection), on day 15 and at the experimental endpoint. **d**, Luminescence curves of each individual mouse. **e**, Survival curves of mice from **c** and **d** (*χ*^2^ = 31.62, log rank < 0.0001). **f**, Flow-cytometry panels showing MOLM-14 cells (BC8^+^mCherry^+^) pre-gated on human cells (BC8^+^mCD45^−^) (top row) in the BM of mice that were humanized with either unedited or edited HSPCs (sgRNA–NTC or sgRNA-49.3) and subsequently treated with either saline solution or CIM053–SG3376. Edited (BC8^+^QA17A19^−^) and unedited (BC8^+^QA17A19^+^) cells in the non-tumour human-cells population (BC8^+^mCherry^−^, green gate) are also shown (bottom). Absolute cell counts of representative panels calculated according to input counting beads are written below each panel. **g**, Quantification of MOLM-14 cells (BC8^+^mCD45^−^mCherry^+^, left) and non-tumour human cells (BC8^+^mCD45^−^mCherry^−^, right) in all mice from all treatment groups. Selected *P* values are shown. In **c**–**g**, 3–4 mice in saline groups and 10 mice in CIM053–SG3376-treated groups; data are mean ± s.d. Ordinary two-way ANOVA tests (*α* = 0.05) were used to assess the statistical difference between groups. Source Data

**Extended Data Fig. 7:**
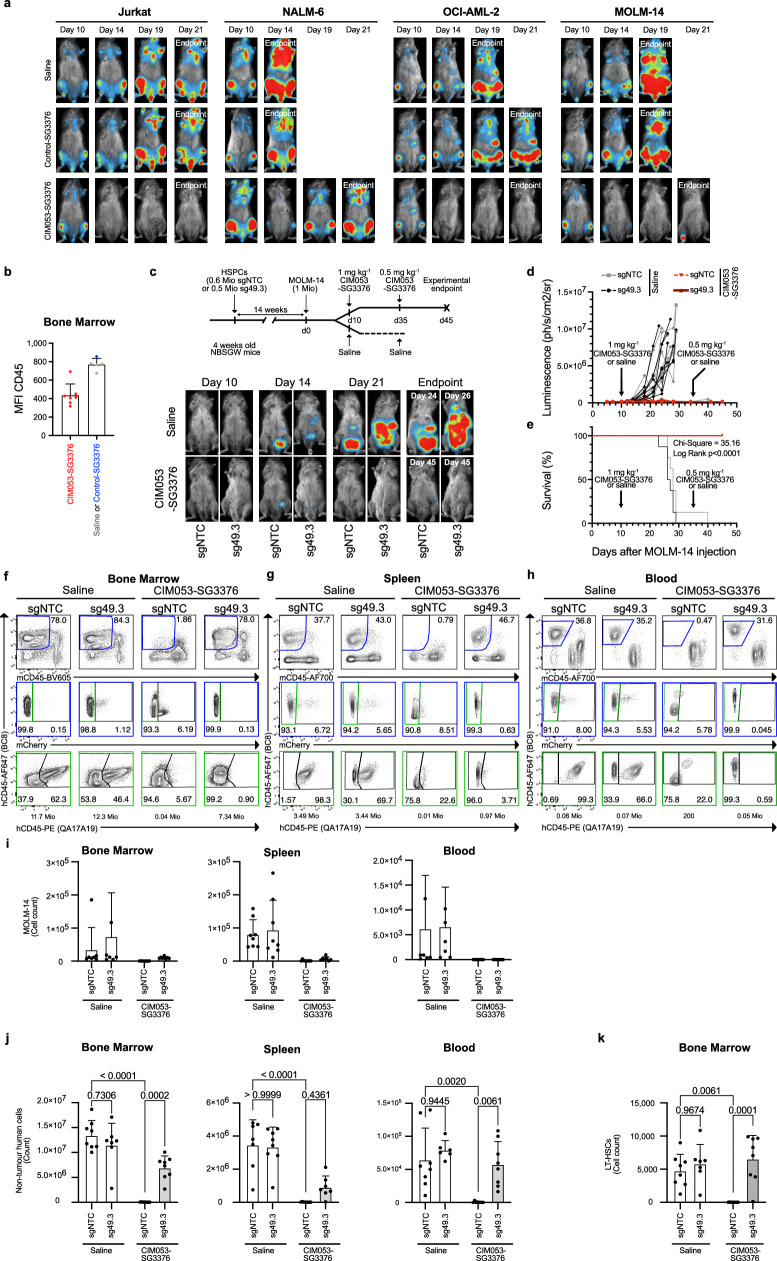
In vivo CIM053-SG3376 mediated tumour treatment with preserved haematopoiesis of edited cells. **a**, Images depicting representative mice for each tumour model (Jurkat, NALM-6, OCI-AML-2 and MOLM-14) before treatment (day 10 post tumour injection), day 14, 19 and 21 (endpoint) for each treatment arm (n = 5 mice for saline, 2 for control-SG3376 and 5 for CIM053-SG3376). **b**, CD45 MFI quantification of bone marrow cells of mice injected with NALM-6 cells and treated with saline, control-SG3376 or CIM053-SG3376 at endpoint. **c**, Experimental timeline of NBSGW mice humanised with control (sgNTC) or shielded (sg49.3) HSPCs, followed by MOLM-14 tumour xenograft injection. Mice were subjected to saline or CIM053-SG3376 treatment. Lower panels: images of representative mice before treatment (day 10 post MOLM-14 injection), day 14, 21, and at endpoint. **d**, Luminescence curves of individual mice. **e**, Survival curve from (**c-d**). Bone marrow (**f**), spleen (**g**) and blood (**h**) flow cytometry panels showing human chimerism (%BC8^+^mCD45^−^, blue gate) of mice humanised with control (sgNTC) or edited (sg49.3) HSPCs and treated with saline or CIM053-SG3376. MOLM-14 cells (BC8^+^mCherry^+^) pre-gated on human cells (%BC8^+^mCD45^−^, blue gate) are displayed by the middle panels. Edited (%BC8^+^QA17A19^−^) and unedited (%BC8^+^QA17A19^+^) cells within the non-tumour human population (%BC8^+^mCherry^−^, green gate) are shown in the bottom panels. Absolute cell counts of representative panels calculated according to input counting beads are written below each panel. **i**, Quantification of MOLM-14 cells (%BC8^+^mCD45^−^mCherry^+^) in bone marrow, spleen and blood. **j**, Quantification of non-tumour human cells (%BC8^+^mCD45^-^mCherry^−^). **k**, Quantification of long-term haematopoietic stem cells (LT-HSC) (%BC8^+^mCD45^−^CD34^+^CD38^−^CD90^+^CD45RA^−^) in the bone marrow. (In all panels, 6-7 mice in saline groups and 8 mice in CIM053-SG3376 treated groups, plotting mean values +/− SD). (Ordinary two-way ANOVA tests (alpha 0.05) were employed to assess statistical difference between groups). Source Data

To test the potential of CIM053–SG3376 to treat tumours, we inoculated mice with the four cell lines assessed in vitro, representing major lineages (Fig. 4a and Extended Data Fig. 5e). All tumours shrank rapidly after a single intravenous dose of CIM053–SG3376, whereas tumours treated with saline or a SG3376-conjugated control mAb expanded exponentially (Fig. 4b and Extended Data Fig. 7a). However, NALM-6 tumour cells relapsed in all mice despite almost complete elimination of the initial extensive disease burden. Relapsing cells displayed reduced CD45 expression compared with control treated cells (Extended Data Fig. 7b). By contrast, Jurkat, OCI-AML-2 and most of the MOLM-14 recipients (three out of five) remained tumour free three weeks after tumour implantation (Fig. 4b). These data indicate that CD45-targeting ADCs could have broad applications, especially for AML and T-cell malignancies, for which the medical need among the haematological cancers is highest. We therefore chose MOLM-14 for follow-up experiments, because it is an aggressive myeloid leukaemia cell line that is frequently used to preclinically assess new immunotherapies for AML in mouse models^11^. Furthermore, the occurrence of a small relapse in a few of the treated mice provided the opportunity to investigate whether repetitive CIM053–SG3376 administration could treat or prevent MOLM-14 relapse (Fig. 4b and Extended Data Fig. 7a).

To examine whether tumours could be targeted selectively without affecting shielded haematopoietic cells, NBSGW mice were xenografted with ^sgNTC^HSPC or ^sg49.3^HSPCs for 14 weeks. Then, MOLM-14 cells were injected, followed by saline or two CIM053–SG3376 doses on days 10 and 35 (Extended Data Fig. 7c). Tumours grew rapidly in both saline groups and necessitated euthanasia owing to weight loss (Extended Data Fig. 7c–e). By contrast, tumours became undetectable in CIM053–SG3376-treated mice. One mouse had a localized relapse on day 34 that disappeared after the second CIM053–SG3376 dose. Thereafter, all mice remained healthy and tumour free, resulting in survival up to day 45, when the experiment was terminated to analyse the haematopoietic compartment (Extended Data Fig. 7d,e). mCherry^+^ MOLM-14 tumour cells were detectable in saline-treated mice in all examined organs, whereas tumour cells were depleted by CIM053–SG3376 (Extended Data Fig. 7f–i). In addition to tumour cells, the ADC eliminated all unedited hCD45^+^ non-tumour cells (Extended Data Fig. 7f–h,j). Notably, ^sg49.3^HSPCs and their progeny persisted in all tested organs, and all remaining non-tumour human cells were shielded (Extended Data Fig. 7f–h,j). Furthermore, unedited LT-HSCs were completely depleted by CIM053–SG3376 but shielded LT-HSCs resisted (Extended Data Fig. 7k).

**Extended Data Fig. 8:**
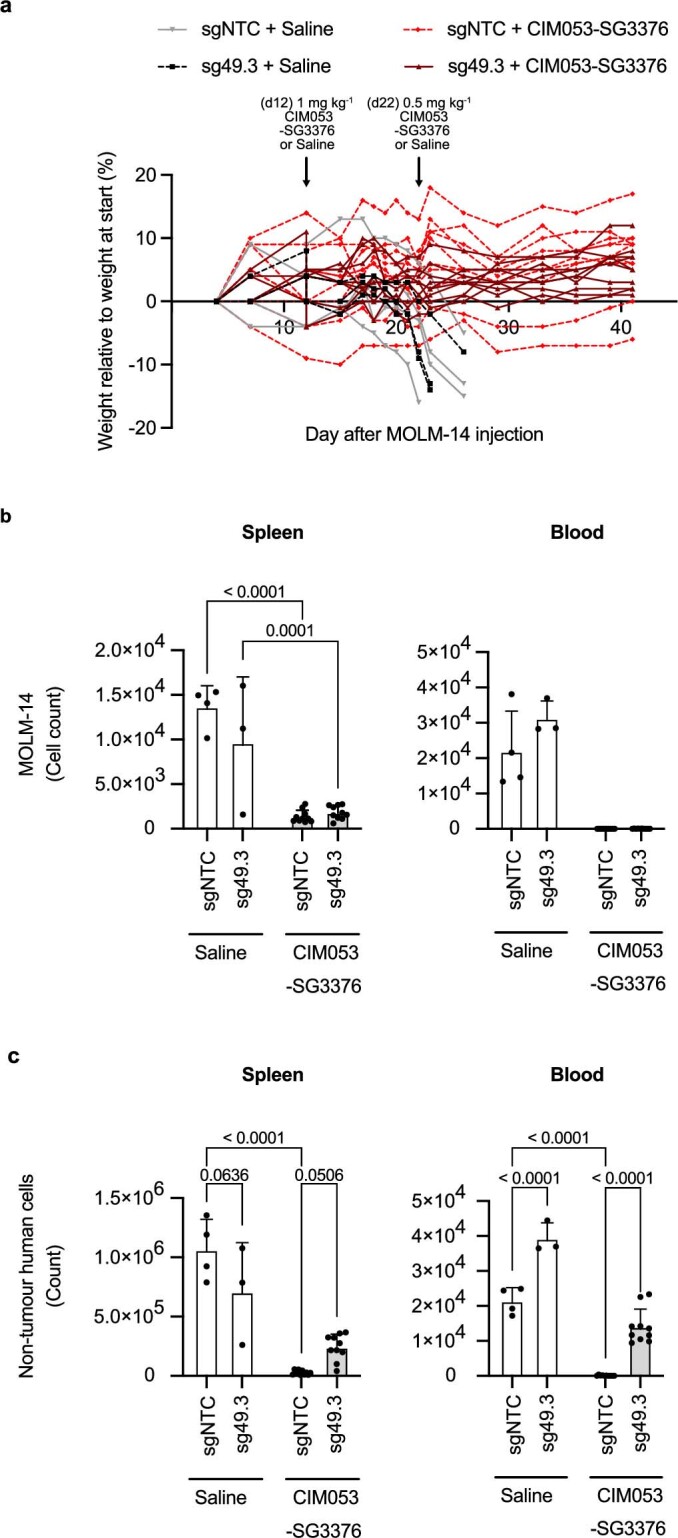
In vivo CIM053-SG3376 mediated selective tumour eradication with preserved haematopoiesis of edited cells. **a**, Weight curves depicting weight loss relative to weight at start of NBSGW mice humanised with control (sgNTC) or shielded (sg49.3) HSPCs, followed by MOLM-14 tumour xenograft injection. The mice were subsequently subjected to either saline treatment or CIM053-SG3376 treatment. **b**, Quantification of MOLM-14 cells (%BC8^+^mCD45^−^mCherry^+^) in the spleen and blood of all mice from (**a**). **c**, Quantification of non-tumour human cells (%BC8^+^mCD45^−^mCherry^−^) in the spleen and blood of all mice from (**a**). (In (**a-c**), 3-4 mice in saline groups and 10 mice in CIM053-SG3376 treated groups, plotting mean values +/− SD). (Ordinary two-way ANOVA tests (alpha 0.05) were employed to assess statistical difference between groups). Source Data

Next, we sought to investigate the therapeutic potency of CIM053-SG3376 for more-advanced disease. CIM053–SG3376 treatment was initiated later and, in an attempt to prevent any relapse, the interval to the second dose was shortened (Fig. 4c, top). Mice showed a more-extensive tumour burden than in the previous experiment (Extended Data Fig. 7c, bottom) and we deliberately assigned the most advanced disease to the CIM053–SG3376 group (Fig. 4c, bottom). Despite this intended bias, CIM053–SG3376 rapidly cleared the tumours in all treated mice and no relapse occurred. By contrast, MOLM-14 grew rapidly in both saline groups, requiring euthanasia of these mice by day 23 after tumour implantation (Fig. 4c–e and Extended Data Fig. 8a). All mice assigned to the ADC groups remained in good health to day 43, when the experiment was terminated (Fig. 4d,e). This finding confirmed that CIM053–SG3376 was well tolerated without overt signs of toxicity, such as weight loss (Extended Data Fig. 8a), and effectively eradicated even advanced disease. Importantly, CIM053–SG3376 simultaneously eliminated unshielded non-tumour human cells but did not affect healthy shielded haematopoiesis (Fig. 4f,g and Extended Data Fig. 8b,c). Collectively, these data demonstrate that CIM053–SG3376 is a highly potent CD45-targeting ADC. Combining it with ^sg49.3^HSPCs enabled tumour-selective immunotherapy in an aggressive AML model and allowed the preservation of a molecularly shielded haematopoietic system.

## Eradication of patient-derived AML in vivo

**Fig. 5:**
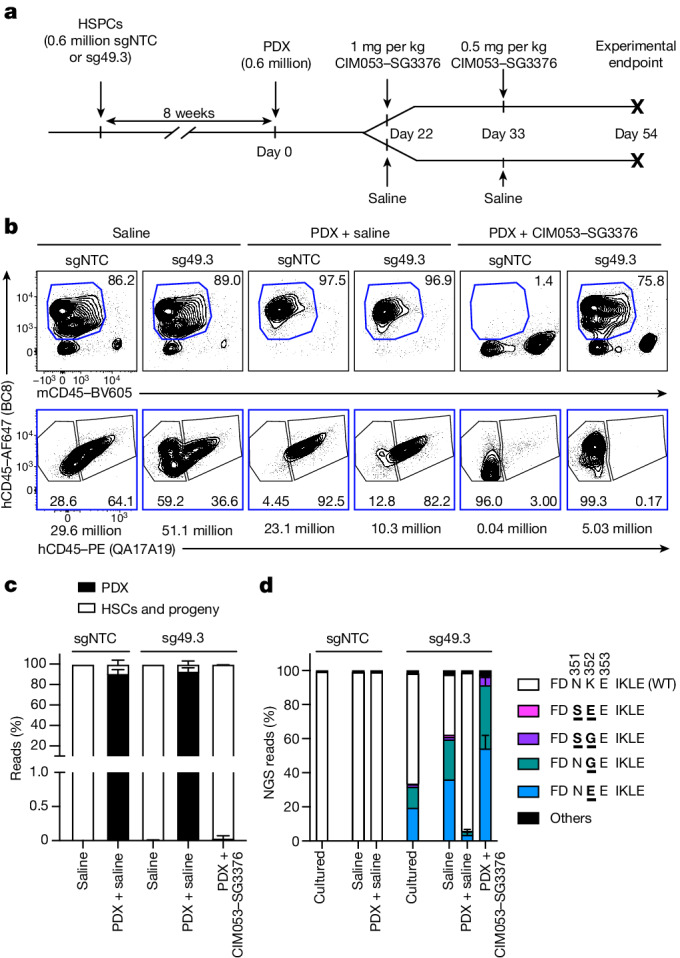
In vivo CIM053–SG3376-mediated selective PDX eradication with preserved haematopoiesis of edited cells. **a**, Experimental timeline of NBSGW mice (4 weeks old) humanized with control (sgNTC) or edited (sg49.3) HSPCs, followed by PDX injection. Mice were subsequently treated with saline or CIM053–SG3376. **b**, Flow-cytometry images (top) showing the percentage of human chimerism (BC8^+^mCD45^−^, blue gate) in the BM of mice at the experimental endpoint. Edited (BC8^+^QA17A19^−^) and unedited (BC8^+^QA17A19^+^) cells in the human-cell population (BC8^+^mCD45^−^, blue gate) are also shown (bottom). Absolute cell counts of representative panels calculated according to input counting beads are below each panel. **c**, Quantification of human chimerism (PDX versus HSC donor-derived cells) in the BM of all mice. **d**, NGS of matching electroporated cells cultured in vitro and from the BM of all animals. NGS reads accounting for less than 0.8% of total reads were classified as ‘others’. In **b**–**d**, data are from 1–6 mice depending on the group. Data are mean ± s.d. Ordinary two-way ANOVA tests (*α* *=* 0.05) were used to assess statistical differences between groups. Source Data

**Extended Data Fig. 9:**
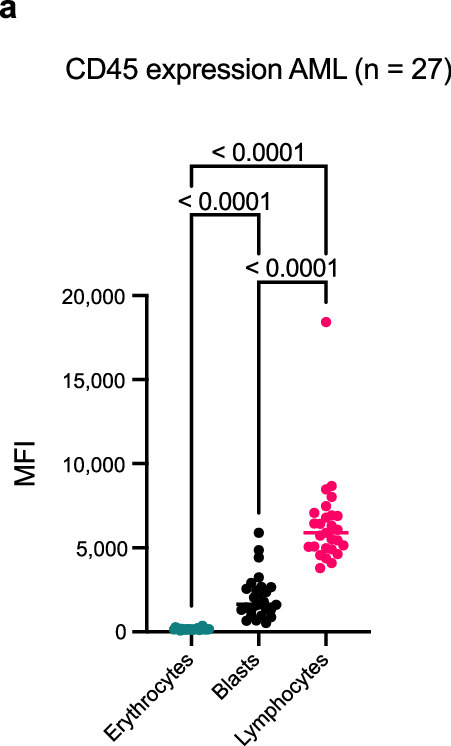
CD45 expression in AML patient samples at primary diagnosis. **a**, CD45 mean fluorescence intensity (MFI) of CD45 of AML blasts, erythrocytes (negative control) and lymphocytes (positive control) of 27 AML patients assessed at initial diagnosis. (Tukey’s multiple comparisons test was employed to assess statistical difference between groups. The statistical significance is displayed with mixed-effects analysis (REML)).

**Extended Data Fig. 10:**
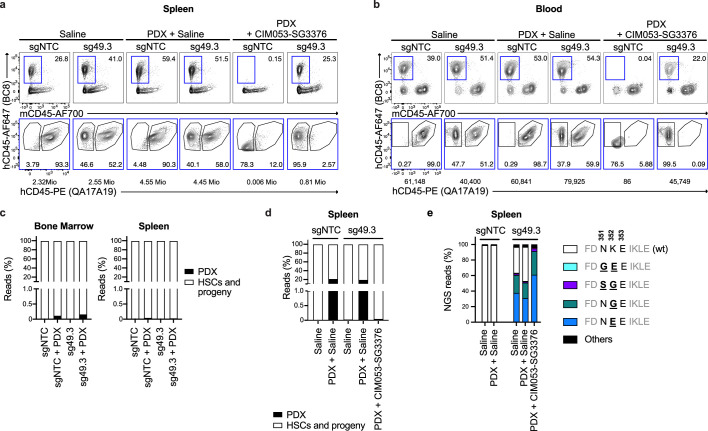
In vivo CIM053-SG3376 mediated selective PDX eradication with preserved haematopoiesis of edited cells. **a**, Flow cytometry analysis depicting the percentage of human chimerism (%BC8^+^mCD45^−^, blue gate) in the spleen (**a**) and blood (**b**) of mice humanised with control (sgNTC) or edited (sg49.3) HSPCs and subsequently treated with either saline solution or CIM053-SG3376. Edited (%BC8^+^QA17A19^−^) and unedited (%BC8^+^QA17A19^+^) cells within the human cell population (%BC8^+^mCD45^−^, blue gate) are also shown (bottom panels). Absolute cell counts of representative panels calculated according to input counting beads are written below the plot. **c**, Quantification of the human chimerism (PDX versus HSC donor derived cells) in the bone marrow and spleen of mice humanised with control (sgNTC) or shielded (sg49.3) HSPCs, followed by PDX injection, 3 days before saline or CIM053-SG3376 treatment (1-3 mice per group). **d**, Quantification of the human chimerism (PDX vs HSC donor derived cells) in the spleen of mice from (Fig. 5c). **e**, Next generation sequencing of spleens of mice from (Fig. 5c). NGS reads accounting for less than 0.8% of total reads were classified as “Others”. (In (**a, b, d, e**), 1-6 mice depending on the group, data are presented as mean values). Source Data

Finally, we assessed CD45 expression on AML samples at primary diagnosis and investigated the potency of CIM053–SG3376 on patient-derived AML grafts. Analysis of a previously published single-cell RNA sequencing dataset showed that CD45 transcripts are expressed more homogeneously in AML cells than the more established AML targets CD33, CD123 and FLT3 (ref. ^31^). Furthermore, analysis of AML blasts by flow cytometry includes gating on CD45^dim^ cells; that is, AML cells express CD45 by definition. To quantify CD45 expression on AML blasts (from 27 people with AML) that were analysed at primary diagnosis, we used erythrocytes as a negative denominator and confirmed substantial CD45 expression in all samples (Extended Data Fig. 9a). Lymphocytes that expressed high CD45 copy numbers^22^ displayed even higher expression. To test selective AML depletion with patient-derived cells, we engrafted AML patient-derived xenograft (PDX) cells in mice that were previously xenografted with ^sgNTC^HSPCs or ^sg49.3^HSPCs. Subsequently, we used two intravenous doses of CIM053–SG3376 (Fig. 5a). Engraftment of PDX altered the flow cytometry profile of human–mouse chimerism in BM (Fig. 5b, top) but not in spleen or blood (Extended Data Fig 10a,b). However, AML cells could not directly be distinguished from CD45^wt^ HSPC-derived cells because we used unlabelled PDX and both express CD45^WT^. Even so, injecting PDX reduced the relative number of shielded cells (BC8^+^QA17A19^−^) in BM from 59.2% to 12.8%, but there was no such reduction in spleen or blood, indicating that PDX cells displaced ^sg49.3^HSPC-derived cells in BM (Fig. 5b, bottom). In support of this, CIM053–SG3376 administration depleted all hCD45^+^ cells in mice engrafted with ^sgNTC^HSPCs, but ^sg49.3^HSPC-derived hCD45^+^ cells persisted and the ADC treatment restored the flow cytometry profile seen in mice without PDX and slightly increased mouse CD45^+^ cells (Fig. 5b, top). Importantly, CIM053–SG3376 treatment resulted in a pure population of edited hCD45^+^ cells in all organs, indicating that PDX cells were eliminated and only shielded cells remained (Fig. 5b, bottom, and Extended Data Fig. 10a,b). To verify this directly, we used genetic chimerism analysis to discriminate between PDX and HSPC-derived cells. Shortly before CIM053–SG3376 treatment, PDX cells were detectable in BM but not in spleen (Extended Data Fig. 10c). At the endpoint, more than 90% of BM and 20% of spleen reads were PDX, unequivocally confirming PDX infiltration in control mice and PDX eradication by CIM053–SG3376 (Fig. 5c and Extended Data Fig. 10d). This was further confirmed by NGS of on-target editing (Fig. 5d and Extended Data Fig. 10e). Editing frequencies were similar to those in the previous experiment without tumour injection (Fig. 3g). However, reads for on-target editing were reduced to less than 10% in mice xenografted with ^sg49.3^HSPC plus PDX (saline), confirming that PDX cells infiltrated BM and displaced ^sg49.3^HSPC-derived haematopoiesis. In line with the flow cytometry data, CIM053–SG3376 administration yielded a pure population of edited cells. Collectively, these results demonstrate the complete clearance of PDX cells with preserved shielded haematopoiesis.

## Discussion

Here, we have shown that CD45 constitutes an excellent target for antigen-specific therapy of haematological diseases. Although CD45 has long been a pharmacological target of interest^32^, its importance for haematopoietic cells and broad expression precluded continuous treatment with a depleting drug. Combining a potent CD45-targeting ADC with shielded HSCs generated a large therapeutic window, which solved the problem by enabling continued post-transplantation therapy and radical, but well tolerated, selective tumour eradication. Our data further demonstrate that a CD45-targeting ADC broadly cleared an individual’s haematopoietic cells, including HSCs, indicating that it could condition patients before HSCT, avoiding systemic side effects caused by untargeted conventional conditioning^12^. Because CD45 is expressed by most haematological cancers, including AML, the same drug could simultaneously condition for HSCT and target most cancer cells independent of their origin.

We and others have independently demonstrated the potential of epitope engineering for therapeutic applications^18–21^. However, the emerging data also underline the importance of carefully choosing and characterizing the depleting agent and the target protein variants, and selecting the most suitable genome-editing approach. For instance, residual binding resulted in incomplete shielding from a CAR T (CD123^S59Y^)^19^, and the unintended BE bystander CD45^H285R^ negatively affected the intended CD45^N286D^ shielding variant (Fig. 1d,e). Thus, the desired amino acid substitution and genomic context influence the choice of the most appropriate genome-editing approach. We previously used CRISPR/Cas9-mediated homology-directed repair to install single amino acid substitutions to serve as molecular shields^18,19^. Here, we used a BE that was efficient and well tolerated by HSPCs. However, despite the favourable characteristics of the BE, such as the purported avoidance of DNA double-strand breaks and aptness for multiplexing^23,33^, we discontinued most base-editable CD45 shielding candidates for various reasons. Similarly, we found that two previously described base-editable shielding variants^20,21^ were functionally impaired (CD123^S59P^)^19^ or biophysically suboptimal (CD45^Y232C^). These examples illustrate the most relevant challenges of applying a BE for cell shielding: the enzymatic activity of the BE (cytosine or adenine deaminases) inherently constrains it to defined base conversions (ABE: A > G; CBE: C > T), which severely limits the number of possible amino acid substitutions. Nevertheless, using existing tools we successfully identified a BE/sgRNA that did not affect either the biophysical properties of CD45 or HSPC biology but provided the desired protection from a highly potent cell depleter. In future, prime editing may enable the engineering of a preferred substitution without bystander edits^34^. Thus, the most suitable editing approach should be evaluated on a case-by-case basis^23^.

Let us consider the limitations of this work. Initial off-target analysis revealed some intergenic and intronic off-target editing. However, risk assessment needs to take into account the proven genotoxic effects of the current standard of care (chemotherapy) that results in an increased rate of leukaemia^35^. Given the important medical need to improve the risk–benefit trade-off of HSCT and CD45-targeting ADC being highly promising to achieve this goal^36^, we consider that the off-target editing found here constitutes a reasonable safety profile if used for AML. Nevertheless, on- and off-target editing should be improved further through optimized manufacturing, higher fidelity, more PAM-restricted and/or engineered BE with an altered BE window and/or the use of hybrid RNA–DNA guides^23,25,29,30,37^. A clinical candidate BE should then undergo a more complete safety analysis, for example including HSPCs from more donors, analysis of sgRNA-independent off-target editing, investigation of structural genomic aberrations, including chromosomal translocations, as well as dedicated tumorigenicity studies^38^. Furthermore, although we did not observe any toxicity in this study, inherent ADC limitations, including the risk of off-target toxicity resulting from premature payload release, fast clearance as a result of a high drug:antibody ratio and drug resistance, need to be considered before clinical translation. ADC toxicity-mitigation strategies include a non-cleavable linker, as used in this study, and site-specific linker–payload conjugation methods^39^. Furthermore, cell shielding enables dose fractionation, which should be optimized to reduce peak and cumulative ADC doses. The vast number of cells that express CD45 may result in dose-limiting toxicity in humans as a result of massive cell lysis by the CD45-targeting ADC. However, such toxicity was not observed in immunocompetent mice targeted by anti-murine CD45-targeting ADCs^12,36,40,41^. Clinically, the potential risk may be alleviated by tumour debulking using conventional chemotherapy or patient pretreatment with an epitope-blocking non-depleting antibody similar to obinutuzumab pretreatment in anticipation of glofitamab treatment^42^. Finally, tumour relapse could occur, as with any monotherapy, particularly for AML^39,43^. If antigen escape or drug resistance occur, it may be necessary to administer donor lymphocyte infusions for consolidation. Alternatively, multi-target immunotherapy with multiplex shielded HSPCs^20^ may overcome any resistance.

In summary, the convergence of major progress in different fields enabled a radically new approach to replacing the haematopoietic system. If successfully translated, this strategy has the potential to be used for other diseases that benefit from depleting lymphocytes and replacing a diseased immune system, such as severe autoimmune diseases^44^ and maybe even HIV infection. We predict that the approach presented here not only will enable cancer-selective therapy with preserved function of the haematopoietic system, but could more generally pave the way for synthetic haematopoiesis.

## Materials and Methods

### Structural dataset and computational analysis

The experimentally determined 3D structure of the CD45 extracellular domain was retrieved from the PDB (5FMV)^22^. The per-residue relative solvent accessibility area was computed using a previously published algorithm^45^ implemented in FreeSASA^46^ using default parameters. Prediction of B-cell epitopes was based on BepiPred-2.0 (ref. ^47^) using the default threshold (0.5) for epitope residues nomination. The EV mutation sequence analysis framework^48^ was used to search for the CD45 sequence with the non-redundant UniProtKB database^49^. A multiple-sequence alignment was built using five iterations of the jackhammer HMM search algorithm^50^ with default significance score for the inclusion of homologous sequences.

### Plasmid cloning

For sgRNA cloning into the px458 host vector (a gift from F. Zhang) (Supplementary Table 1), forward and reverse primers containing complementary CRISPR RNA (crRNA) sequences flanked by BbsI restriction sites were used (Supplementary Table 4). The px458 plasmid was double digested with AgeI-HF (NEB, R3552S) and EcoRI-HF (NEB, R3101S) to eliminate the regions coding for GFP and Cas9. The px458 vector was then digested using BbsI (ThermoFisher Scientific, ER1012), gel purified and ligated with the phosphorylated and annealed crRNA oligonucleotides (called sgRNA plasmid once cloned).

To transiently overexpress CD45 mutants, we introduced each variant of interest in a plasmid expressing WT CD45RO. Briefly, we digested the pCD45RABC plasmid (Sino Biological) (Supplementary Table 1) with HindIII-HF (NEB, R3104S) and XcmI (NEB, R0533L) to remove the alternative spliced exons A, B and C. The point mutations of the human CD45 variants were then introduced into the plasmid expressing CD45RO using PCR (Supplementary Table 4).

### Plasmids

All ligations were transformed in JM109-competent bacteria (Promega, P9751). BE plasmids were from Addgene (SPACE-NG, ABE8e-NG, ABEmax-SpRY, ABEmax-SpG, CBE4max-NG, CBE4max-SpG and xCas9(3.7)-BE4) (Supplementary Table 1).

### BE mRNA and sgRNAs

ABE8e-NG mRNA (capped (cap 1) using CleanCap AG; fully substituted with 5-methoxy-U; 120A polyA tail) and ABE8e(TadA-8e V106W)-SpRY mRNA (capped (cap 1) using CleanCap AG 3′-O-methylation; fully substituted with N1-methyl-pseudo-U; 80A polyA tail) were from Trilink Biotechnologies and Tebu-bio. We used 100-base lyophilized chemically modified sgRNAs from Synthego using their CRISPRevolution sgRNA EZ Kit service and resuspended at 100 µM (3.2 µg µl^−1^) in 1× TE buffer from Synthego (10 nM Tris, 1 mM EDTA, pH 8.0; chemical modifications include 2′-O-methylation of the three first and last bases and 3′ phosphorothioate bonds between the first three and last two bases of each sgRNA).

### Genomic DNA extraction, PCR and Sanger sequencing

Cells from the BE plasmid screening were lysed in tail lysis buffer (100 mM Tris pH 8.5, 5 mM Na-EDTA, 0.2% SDS, 200 mM NaCl) containing proteinase K (Sigma-Aldrich) at 56 °C (1,000 rpm) for 1 h. The DNA was precipitated with isopropanol (1:1 volume ratio) and washed in 70% ethanol. The DNA was then resuspended in H_2_O and the genomic DNA concentration was measured with a NanoDrop device (Thermo Fisher).

For samples containing few cells, genomic DNA was extracted using QuickExtract (Lucigen, QE09050). Cell pellets were resuspended in 30 µl QuickExtract, incubated at 60 °C for 6 min, vortexed for 1 min and subsequently re-incubated at 98 °C for 10 min.

PCR was performed using GoTaq G2 Green Master Mix (Promega, M782B). The gDNA of samples analysed by NGS was extracted using QuickExtract (Lucigen, QE09050) or the Quick-DNA 96 Plus kit (Zymo, D4070) and the genomic DNA concentration was measured with a Qubit device (Thermo Fisher).

For Sanger sequencing, different PCR primers were used depending on the CD45 exon targeted by the sgRNA and the sequencing technology (Supplementary Table 4). Sequencing of PCR amplicons was done at Microsynth and sequencing chromatograms were analysed using the EditR R package^51^ to quantify BE efficiencies.

### Next-generation amplicon sequencing

For NGS, targeted amplicon libraries were generated using a three-step PCR protocol. In brief, nested PCRs were done on genomic DNA samples using KAPA HiFi HotStart polymerase (Roche) (Supplementary Table 4). After Illumina barcoding (Nextera indices, Illumina) using KAPA HiFi HotStart polymerase (Roche), PCR samples were pooled, purified using AMPure XP beads (Beckman Coulter) and quantified using Qubit dsDNA HS assay kit (Thermo Fisher). Libraries were paired-end sequenced on an Illumina Miniseq instrument using the Illumina Miniseq Mid output kit (300 cycles) with 50% PhiX spike-in (Illumina). After demultiplexing, each sample was assessed for quality using FastQC^52^ and processed using the CRISPResso2 tool^53^. For each of the samples, we provided the reference amplicon sequence (hg38) and the guide RNA sequence (reverse complement) and defined the quantification window centre to −10, the quantification window size to 15 and the plot window size to 30. We applied minimum paired end reads overlap between 10 and 200 and provided the following Trimmomatic sentence: ILLUMINACLIP:NexteraPE-PE:2:30:10 LEADING:3 TRAILING:3 SLIDINGWINDOW:4:15 MINLEN:36. Finally, we used a custom R script (https://gitlab.com/JekerLab/cd45_shielding) to count and translate into amino acid each allele from the CRISPResso2 output file Alleles_frequency_table.txt in the quantification window. Alleles with less than 0.8% frequency were considered as ‘other’.

### Genetic determination of human chimerism

Genetic discrimination between PDX- and HSPC-derived cells was performed using the Devyser Chimerism NGS kit (Devyser) according to the manufacturer’s recommendations. In brief, sequencing libraries were prepared from genomic DNA targeting 24 polymorphic insertion–deletion markers distributed on 16 different chromosomes. The libraries were sequenced on a MiniSeq (Illumina) instrument using the high-throughput flow cell, generating 74-bp pair-end reads. An informative marker set was defined for the PDX donor/HSPC donor pair. It consisted of 10 markers that reliably discriminated between DNA from PDX and HSPC donor cells. The average proportion of leukaemia donor-specific reads to total reads was calculated to determine the proportion of PDX cells in each sample. It was confirmed that the genomic DNA of the mouse host did not interfere with the analysis.

### CHANGE-seq-BE

Genomic DNA was extracted from human peripheral blood mononuclear cells (PBMCs) using the Puregene tissue kit (Qiagen, 158063) according to the manufacturer’s instructions including the proteinase-K and RNase steps (Qiagen, 158143 and 158153). CHANGE-seq-BE was adapted and modified from the original CHANGE-seq method^54^ to validate genome-wide activity for ABEs^28^. Similar to CHANGE-seq, purified genomic DNA tagmented with a custom Tn5-transposome to generate an average length of 650 bp and followed by gap repair with Kapa HiFi HotStart Uracil + DNA Polymerase (KAPA Biosystems, KK2802) and *Taq* DNA ligase (NEB, M0208L). Gap-repaired DNA was treated with USER enzyme (NEB, M5505L) and T4 polynucleotide kinase (NEB, M0201L). Intramolecular circularization of the DNA was performed with T4 DNA ligase (NEB, M0202L) and residual linear DNA was degraded by a cocktail of exonucleases containing plasmid-safe ATP-dependent DNase (Lucigen, E3110K), lambda exonuclease (NEB, M0262L), exonuclease I (NEB, M0293L) and exonuclease III (NEB, M0206L). The circularized DNA was then treated with Quick CIP (NEB, M025L) to dephosphorylate 5′ and 3′ ends of any residual linear DNA. Circularized genomic DNA (125 ng) was treated with ABE8e–SpRY:sgRNA-49.3 complexes in vitro in a 50 μl reaction for 24 h at 37 °C. ABE RNP complexes nicked the targeted DNA strand and deaminated adenine bases to inosine in the non-targeted stranded DNA of both on- and off-target sites. Further enzymatic steps were included with ABE treatment in the CHANGE-seq-BE method to generate double-strand breaks. Nicked DNA circles were treated with endonuclease V in 10× NEB buffer 4 (NEB, M0305S). Endo V cleaved DNA adjacent to inosines to generate linear DNA with 5′ overhangs. Gaps were filled with klenow fragments (3′ > 5′ exo) and deoxyribonucleotide triphosphates (dNTPs) (NEB, M0212L) in NEB buffer 2. End-repaired DNA products were A-tailed and further ligated with a hairpin adapter using an HTP library preparation kit (Kapa, KK8235), USER treated and amplified by PCR-barcoded universal primers with NEBNext multiplex oligonucleotides for illumina (NEB, E7600S), using Kapa HiFi HotStart uracil master mix. PCR libraries were quantified by quantitative PCR (KAPA Biosystems, KK4824) and sequenced with 151-8-8151 cycles on an Illumina NextSeq 2000 instrument. CHANGE-seq-BE data analyses were performed using open-source software: https://github.com/tsailabSJ/changeseq/tree/dev.

### rhAmpSeq

Validation of off-target sites was performed using the rhAmpSeq system from IDT. rhAmpSeq primer panels for targeted amplification were generated using the rhAmpSeq design tool defining the insert size between 150 and 250 bp. Applied primer sequences are listed in Supplementary Table 4. A rhAmpSeq CRISPR library was prepared according to the manufacturer’s instructions and sequenced on an Illumina MiniSeq instrument (MiniSeq high output kit, 300 cycles). Custom python code and open-source bioinformatic tools were used to analyse rhAmpSeq data. First, we generated FASTQ format files by demultiplexing high-throughput-sequencing BCL data files. Next, the reads were processed using CRISPRessoPooled (v.2.0.41) with quantification_window_size 10, quantification_window_center −10, base_editor_output, conversion_nuc_from A, conversion_nuc_to G. The allele frequency table from the output files was used to calculate the A•T-to-G•C editing frequency. Specifically, the editing frequency for each on- or off-target site was defined as the ratio between the number of reads containing the edited base (that is, G) in a window from positions 4 to 10 of each protospacer (where the GAA PAM is positions 21–23) and the total number of reads. To calculate the statistical significance of off-target editing, we applied a method previously described^55^. In brief, a 2-by-2 contingency table was constructed using the number of edited reads and the number of unedited reads in the treated sample and its corresponding control sample. Next, a *χ*^2^ test was done. The FDR was calculated using the Benjamini–Hochberg method. Significant off-targets were defined on the basis of: first, FDR ≤ 0.05 and second, the difference in editing frequency between treated and control (≥1%).

### Cell line culture conditions

All cancer cell lines (listed in Supplementary Table 5) were cultured in RPMI-1640 (Sigma-Aldrich, R8758) supplemented with 10% heat-inactivated FCS (Gibco Life Technologies) and 2 mM GlutaMAX (ThermoFisher Scientific, 35050061) at 37 °C.

All cell lines were retrovirally transduced with MI-Luciferase-IRES-mCherry (gift from X. Sun; Supplementary Table 1). Cells were then FACS-sorted on the basis of mCherry expression. After expansion, MOLM-14 and OCI–AML2 were profiled for short tandem repeats and tested negative for mycoplasma before being frozen until further use. Jurkat and NALM-6 were purchased from ATCC and were therefore not profiled for short tandem repeats.

DF-1 cells were cultured in DMEM high-glucose medium (Sigma-Aldrich, D5796) supplemented with 10% non-heat-inactivated FCS and 2 mM GlutaMAX at 39 °C (Supplementary Table 5).

### Human primary T cell culture and activation conditions

Leukocyte buffy coats from anonymous healthy human donors were purchased from the blood-donation centre at Basel (Blutspendezentrum SRK beider Basel, BSZ). PBMCs were isolated by density centrifugation using SepMate tubes (StemCell Technologies, 85450) and the density gradient medium Ficoll-Paque (GE Healthcare) according to the manufacturer’s protocol. Frozen PBMCs were thawed and human primary T cells were then isolated using an EasySep human T cell isolation kit (Stemcell Technologies, 17951) following the manufacturer’s protocol. T cells were cultured overnight without stimulation at a density of 1.5 × 10^6^ cells per ml in RPMI-1640 medium supplemented with 10% heat-inactivated human serum (AB^+^, male; purchased from BSZ), 10 mM HEPES (Sigma-Aldrich), 2 mM GlutaMAX, 1 mM sodium pyruvate, 0.05 mM 2-mercaptoethanol and 1% MEM non-essential amino acids (all from Gibco Life Technologies). The next day, the human primary T cells were activated with interleukin-2 (IL-2) (150 U ml^−1^, proleukin, University Hospital Basel), IL-7 (5 ng ml^−1^, R&D Systems), IL-15 (5 ng m^−1^, R&D Systems) and Dynabead Human T-Activator CD3/CD28 (1:1 beads:cells ratio) (Gibco, 11132D). The activated cells were de-beaded before electroporation.

### hCD34^+^ HSPC isolation and culture conditions until electroporation

Leukopaks were purchased from CytoCare and hCD34^+^ HSPCs were isolated by the LP-34 process using CliniMACS Prodigy (Miltenyi). Isolated hCD34^+^ HSPCs were thawed and grown in HSPC medium for two days until electroporation (StemSpan SFEM II (StemCell, 09655) supplemented with 100 ng ml^−1^ human stem cell factor (hSCF) (Miltenyi, 130-096-695), 100 ng ml^−1^ human FMS-like tyrosine kinase ligand (hFlt3)-ligand (Miltenyi, 130-096-479), 100 ng ml^−1^ human thrombopoietin (hTPO) (Miltenyi, 130-095-752) and 60 ng ml^−1^ hIL-3 (Miltenyi, 130-095-069).

### Electroporation conditions

K562 cells (2 × 10^6^) were resuspended in buffer T and mixed with 5 µg BE plasmid (Supplementary Table 1) and 1.5 µg sgRNA plasmid for co-electroporation using a Neon transfection system (ThermoFisher, MPK10096; 1,450 V, 10 ms, 3 pulses). To monitor the electroporation efficiency, GFP expression was evaluated 24 h after electroporation using an optical microscope. In Extended Data Fig. 1f, BE results are displayed using a custom BE score: log((sum of editing frequencies per condition/number of edited positions per condition)+1).

De-beaded human activated T cells (1 × 10^6^) were resuspended in 100 µl Lonza supplemented P3 electroporation buffer with 7.5 µg BE mRNA (1 µg µl^−1^) and 7.5 µg sgRNA (3.2 µg µl^−1^) (Supplementary Table 2) and electroporated using the 4D-Nucleofector system (Lonza) with program EH-115. Immediately after electroporation, 900 µl pre-warmed human T-cells medium was added directly in the cuvettes and incubated for 20 min at 37 °C for the T cells to recover. Cells were then transferred in 48-well flat-bottomed plates (Corning, 3548) and the medium was supplemented with 500 U ml^−1^ IL-2. The medium was renewed every two days.

hCD34^+^ HSPCs (1 × 10^6^) were electroporated 48 h after thawing with 7.5 µg BE mRNA (1 µg µl^−1^) and 13.6 µg sgRNA (3.2 µg µl^−1^) (Supplementary Table 2) with a 1:100 BE:sgRNA molar ratio, following the same protocol as for human T cells but with program CA-137. Electroporated hCD34^+^ HSPCs were kept in culture at 0.5 × 10^6^ cells per ml in a six-well flat-bottomed plate (Corning, 3516) in HSPC medium supplemented with 100 ng ml^−1^ hSCF, 100 ng ml^−1^ hFlt3–ligand and 100 ng ml^−1^ hTPO for in vivo applications and with the addition of 60 ng ml^–1^ hIL-3 for in vitro assays. The medium was renewed every five days. Edited hCD34^+^ HSPCs prepared for in vivo injection were frozen two days after electroporation in cryo-preservation CryoStor CS10 medium (Stem Cell Technologies, 07930) at a density of 10 × 10^6^ cells ml^−1^.

### CFU assays

The CFU assay was started 72 h after gene editing. For each condition, 1.1 ml semi-solid methylcellulose medium (StemCell Technologies) containing 200 cells was plated in a well of a SmartDish (StemCell Technologies, 27370) in duplicates. The cells were incubated at 37 °C, for 14 days. The resulting progenitor colonies were counted and scored using STEMVision Analysis (StemCell Technologies) according to the manufacturer’s instructions. The mean of the total number of colonies in the NTC samples for each experiment was set as 1.

### DF-1 cell-transfection conditions

Plasmid (6.5 µg) encoding WT hCD45RO or its variants was mixed with 200 µl serum-free DMEM medium and 19.5 µl polyethylenimine (1 mg ml^−1^; Chemie Brunschwig, POL23966-100). The transfection mix was added dropwise to 1 × 10^6^ DF-1 cells plated the day before in a six-well plate. Cells were analysed 48 h later by flow cytometry.

### In vitro ADC-mediated killing assays

For in vitro ADC killing assays, 5,000 base-edited human activated T cells or 25,000 base-edited hCD34^+^ HSPCs were plated five days after electroporation in 96-well plates (flat-bottomed for T cells and round-bottomed for HSPCs; Corning 3596 and 3799, respectively) in 100 µl of corresponding medium (supplemented with only 50 U ml^−1^ of IL-2 for human T cells). For ADC killing assays involving saporin, a 100 nM stock was prepared by incubating the biotinylated antibody (BC8 or MIRG451 mAbs) and saporin–streptavidin (ATS-Bio, IT-27-1000) at a 1:1 molar ratio for 30 min at room temperature.

For in vitro ADC killing of co-cultures, 12,500 Jurkat cells were stained for 20 min with CTV (Invitrogen, C34557A) at 37 °C and then seeded at a 1:1 cell ratio with 12,500 base-edited hCD34^+^ HSPCs five days post-electroporation in 96-well round-bottom plates in 100 µl HSPC medium with corresponding concentrations of CIM053–SG3376 (ADC Therapeutics). Cells were incubated for 72 h at 37 °C, stained for flow cytometry or cell sorting and analysed using a BD LSRFortessa. Genomic DNA was extracted for sequencing.

For in vitro ADC killing of mCherry–luciferase-marked tumour cell lines (Jurkat, NALM-6, OCI-AML-2 and MOLM-14), 2,000 cells were plated in 384-well plates in medium with or without 30 min pre-incubation at 37 °C with 50 µg ml^−1^ (333.33 nM) naked CIM053 antibody (40 µl final total volume per well). Following a 72 h incubation period, 5 μl firefly d-luciferin (0.75 mg ml^−1^ resuspended in medium (Biosynth, L-8220)) was added to each well and incubated for 5 min at room temperature. Luminescence readouts were recorded using a BioTek Synergy H1 plate reader.

### Expression and purification of soluble CD45^wt^ and CD45 variants

For precise antibody–protein affinity measurements and biophysical characterization, CD45^wt^ and variants containing only D1 and D2 of the ECD were produced. The protein sequence (residues 225–394) was histidine tagged at the carboxy terminus and contains few N- and C-terminal added amino acids (full WT sequence: ETGIEGRKPTCDEKYANITVDYLYNKETKLFTAKLNVNENVECGNNTCTNNEVHNLTECKNASVSISHNSCTAPDKTLILDVPPGVEKFQLHDCTQVEKADTTICLKWKNIETFTCDTQNITYRFQCGNMIFDNKEIKLENLEPEHEYKCDSEILYNNHKFTNASKIIKTDFGSPGEGTKHHHHHH, SEQ ID 57, Uniprot ID P08575). Expi293F GnTI cells (Thermo Fisher, A39240) that lack *N*-acetylglucosaminyltransferase I (GnTI) activity and therefore lack complex *N*-glycans were used for protein expression. After collection, the protein was purified using Ni-NTA chromatography followed by digestion of high-mannose glycans with endoglycosidase H (EndoHf; New England BioLabs, P0703S) at 37 °C overnight. EndoHf was removed from the protein solution with amylose resin and the CD45 protein was further purified by size-exclusion chromatography in buffer comprising 20 mM HEPES, pH 7.4, 150 mM NaCl. Peak monomer and dimer fractions (where needed) were concentrated using a 10 kDa cut-off Amicon centrifugal filter (UFC8010) and protein aliquots were flash-frozen in liquid nitrogen before storage at −150 °C. Variant CD45 proteins were produced using the same experimental procedure. The monomer content percentage for each protein was taken from the size-exclusion chromatogram.

### Binding analysis of soluble CD45 proteins by BLI

Analysis of MIRG451 and BC8 binding to the selected variants was performed on an Octet system RED96e (Sartorius) or R8 (Sartorius) at 25 °C with shaking at 1,000 rpm using 1× kinetic buffer (Sartorius, 18-1105). The selected variants were screened for their ability to bind to MIRG451 and BC8 using different concentrations of CD45 (WT or variant). MIRG451 was captured by an anti-human Fc-capture biosensor (AHC) (Sartorius, 18-5060) for 300 s at 0.5–1 µg ml^−1^. As an analyte, human CD45^wt^ and variants, containing only domains 1 and 2, were titrated at seven different concentrations, from 1,000 nM to 15.6 nM or from 50 nM to 0.78 nM with a 1:2 dilution series. Association of the analyte to MIRG451 was monitored for 600 s and dissociation of the analyte from MIRG451 was monitored for 1,800 s. Reference subtraction was performed against buffer-only wells. AHC tips were regenerated using 10 mM Gly-HCl, pH 1.7. Data were analysed using the Octet data analysis software HT 12.0. Data were fitted to a 1:1 binding model. Kinetic rates *K*_a_ and *K*_d_ were globally fitted.

To analyse binding to BC8, streptavidin biosensors (Sartorius, 18-5020) were first coated with CaptureSelect biotin anti-LC-κ (murine) conjugate (Thermo Scientific, 7103152100) for 600 s at 1 µg ml^−1^. BC8 was then captured by the coated streptavidin biosensors for 300 s at 0.5–1.0 µg ml^−1^. Analyte titration was performed as for MIRG451. Association of the analyte to BC8 was monitored for 300 s and dissociation of the analyte from BC8 was monitored for 900 s. Reference subtraction, regeneration and data analysis were performed as for MIRG451.

### Characterization of CD45 variants by nanoDSF

The thermostability of CD45 D1–D2 variants was analysed by differential scanning fluorimetry and monitoring tryptophane fluorescence using Nanotemper Prometheus NT.48 NanoDSF or a Nanotemper Prometheus Panta (NanoTemper Technologies)^56–58^. Thermal denaturation was monitored by tryptophane/tyrosine fluorescence at 350 and 330 nm and an excitation wavelength of 280 nm was used. CD45^wt^ and variants were prepared at 0.25–1.0 mg ml^−1^ in 20 mM HEPES, 150 mM NaCl, pH 7.4. Then 10 μl was put into the capillaries and placed into the sample holder. Each protein was measured in triplicates per experiment and the CD45^wt^ was measured in four different experiments. The temperature was increased from 20 °C to 90 °C or 95 °C. The analysis was performed using the ratio of the fluorescent intensities at 350 and 330 nm. The software of the instrument was used to calculate *T*_onset_ and *T*_M_ as well as the mean and s.d. of the triplicates. The melting temperature was determined as the inflexion point of the sigmoidal curve and compared with that of CD45^wt^.

### Flow cytometry analysis and sorting

Flow cytometry was done on BD LSRFortessa instruments with BD FACSDiva software. Data were analysed with FlowJo software. Antibodies used for flow cytometry are listed in Supplementary Table 6. Cells were sorted with BD FACSAria or BD FACSMelody cell sorter instruments. Sorted cells were resuspended in 30 µl QuickExtract. PCRs were performed and sent for Sanger sequencing.

### CD45 expression of AML samples

The CD45 expression of 27 people diagnosed with AML at University Hospital Basel was assessed using routinely acquired flow cytometry data as part of the diagnostic work-up. Gating for AML blasts, lymphocytes and erythrocytes was performed manually using FlowJo 10.10.0. Owing to the experimental set-up (threshold for SSC-A and FSC-A to exclude debris), a distinct erythrocyte population could not be distinguished in all samples (23 of 27). Data were analysed with GraphPad Prism 10 and statistical significance was calculated using mixed-effects analysis. All patients gave written informed consent to the analysis of clinical data for research purposes and the study was approved by the local ethics committee (BASEC-Nr 2023-01372).

### Animal experiments

All animal work was done in accordance with the federal and cantonal laws of Switzerland. Protocols were approved by the Animal Research Commission of the Canton of Basel-Stadt, Switzerland. All mice were housed in a specific pathogen-free condition in accordance with institutional guidelines and ethical regulations. NBSGW (stock 026622) female mice were purchased from Jackson Laboratories. HSPCs were edited as described above. Two days after electroporation, cells were collected and frozen in CryoStor CS10 medium. Cells were thawed on the day of injection, washed and resuspended in PBS. Recipient NBSGW female mice (4 weeks old) were injected intravenously into the tail vein with HSPCs (the number of cells injected varied between 0.6 and 1.1 million and is indicated in each figure). Chimerism was analysed by flow cytometry in blood after ten weeks. Mice were treated with saline or CIM053–SG3376 at the dose(s) and intervals indicated in each figure. For tumour experiments in humanized mice, 1 × 10^6^ MOLM-14–mCherry–luc cells were injected into the tail vein. Then, 10 or 12 days after tumour inoculation, the mice were treated with saline or 1 mg per kg CIM053–SG3376. The mice received a second antibody dose of 0.5 mg per kg CIM053–SG3376 10 or 25 days after the first dose. Mice were euthanized 43 or 45 days after tumour inoculation or when reaching the maximum allowed clinical score. To monitor tumour growth, mice were injected intraperitoneally with 100 μl d-luciferin (BioSynth, L-8220) and were subjected to Newton7.0 imaging (Vilber).

For secondary transplant, NSG–SGM3 female mice (stock 013062) were purchased from Jackson Laboratories. Recipient mice were irradiated the day before the BM transplant with 200 cGy. Primary transplant mice were euthanized, the BM was isolated and 40% of it was re-injected into the new host. Mice from secondary transplants were euthanized 8 weeks after humanization.

MOLM-14–mCherry–luc (1 × 10^6^), OCI-AML-2–mCherry–luc (2 × 10^6^), Jurkat–mCherry–luc (5 × 10^6^) or NALM-6–mCherry–luc (0.5 × 10^6^) cells were injected into the tail vein of NBSGW mice. After tumour inoculation, mice were monitored regularly (for behaviour, weight and imaging). Mice were treated with saline, control-SG3376 or CIM053–SG3376 at the dose and intervals indicated in the relevant figure and euthanized 21 days after tumour inoculation or when reaching the maximum allowed clinical score.

Deidentified patient-derived AML samples were obtained from the PDX repository^59,60^ (Cancer Research Center of Toulouse, France). Signed written informed consent for research use in accordance with the Declaration of Helsinki was obtained from patients and approved by the Geneva Health Department Ethic Committee. PDX cells (0.6 × 10^6^) were injected into the tail vein of humanized NBSGW mice (8 weeks after HSPC injection). The weight of the mice was monitored regularly. Mice were treated with saline or CIM053–SG3376 at the doses and intervals indicated in the figure and the mice were euthanized 54 days after tumour inoculation. Some control mice were euthanized 3 days before antibody treatment.

### Tissue collection and processing

After the mice were euthanized, 0.2 ml blood, both hind legs (femur and tibia) and the spleen were collected from each mouse. Cell suspensions were generated, red blood cells were lysed using ACK lysis buffer and then the cell suspensions were filtered. For tumour experiments, organs were collected on the day of euthanasia, single cell suspensions were generated and frozen in cryo medium. Samples from all mice were thawed and stained on the same day to minimize experimental variability. Cells were stained for different antigens and 30 μl Accucheck counting beads (1,066 microspheres per μl; Invitrogen, PCB100) were added to each sample and the results were analysed by FACS using a BD LSRFortessa instrument.

### Statistics and reproducibility

Statistical analyses were done using GraphPad Prism 9 and 10 software. In all figure legends, *n* refers to the number of experimental replicates. For multiple comparisons, two-way ANOVA tests were used with significance levels indicated. Data are presented as mean ± standard deviation. Survival curves were analysed using the log-rank Mantel–Cox test. rhAmpSeq was analysed using a *χ*^2^ test. The FDR was calculated using the Benjamini–Hochberg method.

Some data points of the in vivo experiments were excluded after visual inspection of samples if the FACS time gate showed irregularities. One mouse that did not engraft HSPCs was excluded from Fig. 5 and Extended Data Fig. 10. Cell numbers in the sgNTC group treated with CIM053–SG3376 were so low that analysis of some assays became unreliable (NGS, genetic chimerism analysis). We therefore excluded this group from NGS.

The number of biological replicates is specified for each experiment in the relevant figure legend. Several key experiments were performed by different people at times in different laboratories, and reagents were shared. For instance, identification of variants, characterization of recombinant variants and FACS validation were performed by different people. Some experiments were performed in the academic lab and validated in Cimeio labs and vice versa. To avoid unconscious bias when assigning mice to saline or the CIM053–SG3376 groups, we always assigned the mice with the largest tumour mass to the ADC group.

The investigator who determined genetic chimerism (NGS and analysis) was blinded and provided the results to the investigator in charge of supervising in vivo experiments. The people who performed CHANGE-Seq_BE, rhAMPSeq and analysed the data were blinded and provided the results to the investigator in charge of supervising the in vivo experiments.

### Availability of materials

Non-proprietary materials are freely available on reasonable request. Restrictions apply to proprietary, commercial material.

### Reporting summary

Further information on research design is available in the Nature Portfolio Reporting Summary linked to this article.

## Online content

Any methods, additional references, Nature Portfolio reporting summaries, source data, extended data, supplementary information, acknowledgements, peer review information; details of author contributions and competing interests; and statements of data and code availability are available at 10.1038/s41586-024-07456-3.

### Supplementary information

Supplementary Information This file contains Supplementary Texts 1 and 2; Supplementary Methods 1-4; Supplementary References and legends to Supplementary Tables.

Reporting Summary

Supplementary Table 1 Plasmids used in this study.

Supplementary Table 2 sgRNAs used in this study.

Supplementary Table 3 Genomic coordinates of the rhAMP-SEQ performed in this study.

Supplementary Table 4 Oligonucleotide primers used in this study.

Supplementary Table 5 Cell lines used in this study.

Supplementary Table 6 Antibodies used in this study.

### Source data

Source Data Fig. 1

Source Data Fig. 2

Source Data Fig. 3

Source Data Fig. 4

Source Data Fig. 5

Source Data Extended Data Fig. 6

Source Data Extended Data Fig. 7

Source Data Extended Data Fig. 8

Source Data Extended Data Fig. 10

## Data Availability

All the data supporting the findings of this study are available in the paper and the Supplementary Information (including source data for main figures and Extended Data Figures reporting mouse data and FACS gating strategies) or public repositories. Datasets for targeted amplicon sequencing, CHANGE-SEQ-BE and rhAmp-Seq are available at the European Nucleotide Archive under the following accession number: PRJEB74081. Source data are provided with the paper.

## Code availability

The code for NGS analysis is available at: https://gitlab.com/JekerLab/cd45_shielding. The code for off-target analysis is available at: https://github.com/tsailabSJ/MKSR_off_targets/blob/main/data/raw_data/crispressoPooled_allele_edit_eff.py.
