# Supplementary material for: Selective haematological cancer eradication with preserved haematopoiesis
Source: Nature. 2024 May 22;630(8017):728–35. doi: 10.1038/s41586-024-07456-3 (PMC11186773; doi:10.1038/s41586-024-07456-3)
Supplement: Supplementary file 1 — This file contains Supplementary Texts 1 and 2; Supplementary Methods 1-4; Supplementary References and legends to Supplementary Tables. [file 41586_2024_7456_MOESM1_ESM.pdf]

nature portfolio

https://doi.org/10.1038/s41586-024-07456-3

Supplementary information

Selective haematological cancer eradication with preserved haematopoiesis

In the format provided by the authors and unedited

Supplementary Information

Selective Haematological Cancer Eradication with Preserved Haematopoiesis

Authors:

Simon Garaudé*, Romina Marone*, Rosalba Lepore, Anna Devaux, Astrid Beerlage, Denis Seyres, Alessandro Dell' Aglio, Darius Juskevicius, Jessica Zuin, Thomas Burgold, Sisi Wang, Varun Katta, Garret Manquen, Yichao Li, Clément Larrue, Anna Camus, Izabela Durzynska, Lisa C. Wellinger, Ian Kirby, Patrick H. Van Berkel, Christian Kunz, Jérôme Tamburini, Francesco Bertoni, Corinne C. Widmer, Shengdar Q. Tsai, Federico Simonetta, Stefanie Urlinger and Lukas T. Jeker†

† Corresponding author: lukas.jeker@unibas.ch
* These authors contributed equally to the work

Table of Contents:

Supplementary Text 1 (describing Extended Data Fig. 1): 2
Supplementary Text 2 (describing Extended Data Fig. 3): 2
Supplementary Methods 1: 2
Supplementary Methods 2: 2
Supplementary Methods 3: 2
Supplementary Methods 4: 2
Supplementary Table 1: 2
Supplementary Table 2: 2
Supplementary Table 3: 3
Supplementary Table 4: 3
Supplementary Table 5: 3
Supplementary Table 6: 3

Supplementary Text 1 (describing Extended Data Fig. 1):

Supplementary text describing technical details of a base editor screen used to generate antibody-discernible amino acid substitutions in the CD45 ECD. Description of selection criteria for base editors/CD45 variants chosen for subsequent experiments.

Supplementary Text 2 (describing Extended Data Fig. 3):

Supplementary text describing base editing-mediated shielding of human primary T cells from the BC8 or MIRG451 antibody. In addition, validation of shielding of edited T cells from a surrogate CD45-ADC (BC8-Saporin).

Supplementary Methods 1:

Flow cytometry gating strategy of ex vivo base edited human T cells (**a**) and HSPCs alone (**b**) or co-cultured with Jurkat cells (**c**). Sorted populations used for Sanger sequencing are depicted (**a, c**). Related to Figure 2, Extended Data Figure 3, 4 and 5.

Supplementary Methods 2:

Flow cytometry gating strategy of bone marrow (**a**), spleen (**b**) and blood (**c**) from mice which received $^{sg49.3}$HSPCs and were treated with saline. Related to Figure 3 and Extended Data Figure 6.

Supplementary Methods 3:

Flow cytometry gating strategy of bone marrow (**a**), spleen (**b**) and blood (**c**) from mice which received $^{sg49.3}$HSPCs, MOLM-14 and were treated with saline. Related to Figure 4, Extended Data Figure 7 and 8.

Supplementary Methods 4:

Flow cytometry gating strategy of bone marrow (**a**), spleen (**b**) and blood (**c**) from mice which received $^{sg49.3}$HSPCs, PDX and were treated with saline. Related to Figure 5 and Extended Data Figure 10.

Supplementary Table 1:

Plasmids used in the study.

Supplementary Table 2:

sgRNAs used in the study.

Supplementary Table 3:

Genomic coordinates of the rhAMP-SEQ performed in the study.

Supplementary Table 4:

Oligonucleotide primers used in the study.

Supplementary Table 5:

Cell lines used in the study.

Supplementary Table 6:

Antibodies used in the study.

Supplementary Text 1 for Extended Data Fig. 1

Identification of base editable residues in the CD45 extracellular domain to provide shielding from antibody binding

We paired the sgRNAs with BEs covering different architectures, e.g. cytosine base editors (CBE) and adenine base editors (ABE) with different PAM restrictions available at the time of the screening (SPACE-NG[1], ABE8e-NG[2], ABEmax-SpRY, ABEmax-SpG, CBE4max-NG, CBE4max-SpG and xCas9(3.7)-BE4[3]) (**Extended Data Table 1**). Combinations of plasmids encoding BEs and sgRNAs were co-electroporated in K562 cells and BE frequencies were quantified from Sanger sequencing using EditR[4] after 3 days **(Extended Data Fig. 1e)**. We established a base editing score (described in the Materials and Methods section) that reflected a cumulative editing activity at all edited positions combined to compare editing performance **(Extended Data Fig. 1f).** ABE8e-NG displayed the highest editing activity followed by ABEmax-SpG and ABEmax-SpRY. ABEs tended to edit more actively and more often than CBEs. Some sgRNAs did not lead to any editing independent of the BE they were paired with (sgRNAs 19, 22, 28, 29, 30, 31 and 48). Conversely, some sgRNAs induced editing with most BEs (sgRNAs 1, 5, 6, 8, 10, 11 and 38). Next, we analyzed the codon changes resulting from the editing and observed a broad range of editing outcomes: single digit editing at unique bases, silent edits or very high editing (>80%) at one or more bases with the potential to change multiple amino acids. To validate initial screening hits, we sorted successfully electroporated GFP$^+$ K562 cells (**Extended Data Fig. 1g**). For several conditions bystander edits undetected from the initial screen were found. In many cases the most active BE (ABE8e-NG) also yielded the broadest editing window (**Extended Data Fig. 1h**). To select candidates for further assessment, we considered the editing efficiencies, editing purity, as well as theoretical physicochemical and structural properties of the inferred amino acid substitutions. For example, ABEmax-SpG + sgRNA-15 generated a highly efficient single base edit, I328V. Although this variant is highly attractive from an editing point of view, it is located slightly outside the identified MIRG451 epitope and the conservative Ile-to-Val mutation very likely

would not fully shield CD45 from antibody binding. Despite high editing efficiencies, some substitutions were excluded from further analysis due to their localization, low surface accessibility and/or their structural role ($CD45^{Y365H}$, $CD45^{I371M}$ or $CD45^{Y365H+C367R}$, $CD45^{C367Y+D368H}$) (**Extended Data Fig. 1a,b**). Finally, sgRNAs 7, 44 and 49 coupled with different base editors generated amino acid substitution profiles altering $CD45^{E259}$ (sgRNA-7, D1), $CD45^{N286}$ (sgRNA-44, D1) and $CD45^{K352}$ (sgRNA-49, D2), found to be key residues of BC8 and MIRG451 epitopes, respectively (**Fig. 1c-e**). Taking all these parameters into account we selected candidates for subsequent experiments.

1 Grunewald, J. *et al.* A dual-deaminase CRISPR base editor enables concurrent adenine and cytosine editing. *Nat Biotechnol* **38**, 861-864 (2020). https://doi.org:10.1038/s41587-020-0535-y
2 Richter, M. F. *et al.* Author Correction: Phage-assisted evolution of an adenine base editor with improved Cas domain compatibility and activity. *Nat Biotechnol* **38**, 901 (2020). https://doi.org:10.1038/s41587-020-0562-8
3 Walton, R. T., Christie, K. A., Whittaker, M. N. & Kleinstiver, B. P. Unconstrained genome targeting with near-PAMless engineered CRISPR-Cas9 variants. *Science* **368**, 290-296 (2020). https://doi.org:10.1126/science.aba8853
4 Kluesner, M. G. *et al.* EditR: A Method to Quantify Base Editing from Sanger Sequencing. *CRISPR J* **1**, 239-250 (2018). https://doi.org:10.1089/crispr.2018.0014

Supplementary Text 2 for Extended data Fig. 3

Base editing to prevent antibody binding to primary cells

Next, we electroporated human T cells with ABE8e-NG delivered as mRNA + sgRNA (**Extended Data Table 4**) to investigate base editing-mediated shielding in primary cells. Flow cytometry confirmed that in comparison to sgRNA-NTC, ABE8e-NG + sgRNA-7 (to generate $CD45^{E259G}$) resulted in 8.8% $BC8^{low}$ cells as well as 0.9% $BC8^{-}$ cells with preserved MIRG451 binding (**Extended Data Fig. 3a**). Sanger sequencing of each sorted cell subpopulation validated on-target editing (Codons for $CD45^{E259}$ base editing outcomes are shown in **Extended Data Fig. 3b)**. Indeed, within $BC8^{+}$ cells we detected some $CD45^{E259G}$ editing, possibly heterozygous cells or representing an impurity due to overlapping populations. In addition, silent $CD45^{E259E}$ was found (**Extended Data Fig. 3c**). $BC8^{low}$ and $BC8^{-}$ cells were nearly completely edited for A_4 to G_4 ($CD45^{E259G}$) while A_5 to G_5 was slightly less frequent ($CD45^{E259E}$). Thus, the up to 96.5% enrichment of G_4 in $BC8^{low}$ cells demonstrated that this edit was limiting to result in reduced BC8 binding while A_5 to G_5 conversion represented an irrelevant bystander edit. ABE8e-NG + sgRNA-44 ($CD45^{I283M+H285R+N286D}$) generated 9.4% $BC8^{low}$ and 13.4 % $BC8^{-}$ cells compared to the sgRNA-NTC (**Extended Data Fig. 3a**). The separation of the $BC8^{-}$ cells generated by sgRNA-44 ($CD45^{I283M+H285R+N286D}$) was very distinct whereas the $BC8^{low}$/$BC8^{-}$ populations generated by sgRNA-7 ($CD45^{E259G}$) were much more overlapping with unedited cells. In accordance with FACS and affinity data (**Fig. 1c,d**), this likely represented residual BC8 binding to $CD45^{E259G}$ in T cells known to strongly express CD45. We did not observe any limiting base conversions as no edited positions were enriched close to 100% in the $BC8^{-}$ population (**Extended Data Fig. 3d,e**). A_8>G_8 ($CD45^{H285R}$) was the dominant base conversion (69.5%) followed by A_{10}>G_{10} ($CD45^{N286D}$) (30.5%) while A_3>G_3 ($CD45^{I283M}$) was found in up to 15.5% of $BC8^{-}$ cells. Thus, the editing outcome with sgRNA-44 ($CD45^{I283M+H285R+N286D}$) was more heterogeneous than for sgRNA-7 ($CD45^{E259G}$) and multiple amino acid substitutions contributed to the reduced BC8 binding. ABE8e-NG + sgRNA-49 ($CD45^{N351D+K352E}$) resulted in 4.6% $MIRG451^{low}$ and 1.1 % $MIRG451^{-}$ cells with preserved BC8

binding compared to sgRNA-NTC (**Extended Data Fig. 3a**). Similar to the cell population generated by sgRNA-44 ($CD45^{I283M+H285R+N286D}$), the $MIRG451^{-}$ cells generated by sgRNA-49 ($CD45^{N351D+K352E}$) formed a distinct population suggesting complete non-binding of MIRG451 in accordance with FACS and affinity data (**Fig. 1c,d**). All $MIRG451^{-}$ cells (99%) contained the A_7>G_7 ($CD45^{K352E}$) conversion while A_5>G_5 ($CD45^{N351S}$) and A_4>G_4 ($CD45^{N351D}$) were found in 42% and 70% of $MIRG451^{-}$ cells, respectively (**Extended Data Fig. 3f,g**). Together, these sequencing results coupled with the variant overexpression (**Fig. 1c**) suggest that, when introduced by the BE tested here, $CD45^{E259G}$ and $CD45^{K352E}$ are the defining amino acid substitutions that reduce or prevent binding of BC8 and MIRG451, respectively. For the $CD45^{I283M+H285R+N286D}$ triple variant, $CD45^{H285R}$ and $CD45^{N286D}$ may cooperate in inducing the BC8 complete loss of binding although each single substitution appears sufficient on their own. $CD45^{H285R}$ occurred most frequently while the $CD45^{I283M}$ bystander edit had no impact on BC8 binding.

Base editing shields primary cells from a CD45 surrogate ADC

To investigate whether the epitope engineered cells were protected from ADC-mediated cytotoxicity, we used BC8-Saporin (BC8-SAP) as a surrogate ADC[1]. We focused on ABE8e-NG combined with sgRNA-7 ($CD45^{E259G}$) and sgRNA-44 ($CD45^{I283M+H285R+N286D}$) since the base editing efficiency with sgRNA-49 ($CD45^{N351D+K352E}$) was too low (**Extended Data Fig. 3a**). Human T cells base edited with sgRNA-7 ($CD45^{E259G}$) or sgRNA-44 ($CD45^{I283M+H285R+N286D}$) were incubated with increasing concentrations of BC8-Saporin (BC8-SAP). 3 days later, the cell composition was analyzed by flow cytometry and live cells were sorted and sequenced. 1nM free SAP did not affect the ratio of edited to non-edited cells (**Extended Data Fig. 3h,i,k,l**). In contrast, $BC8^{+}$ cells were preferentially killed by increasing BC8-SAP concentrations resulting in gradual disappearance of BC8-stained cells (**Extended Data Fig. 3h,i,k,l**) and an enrichment of the base editing frequency (**Extended Data Fig. 3j,m**). $CD45^{E259G}$ base edited cells were shielded from ADC killing up to 0.25nM BC8-SAP. Higher BC8-SAP concentrations resulted in partial killing of the base edited cells, likely due to residual

BC8 binding to CD45^{E259G} (**Extended Data Fig. 3h,i; Fig. 1c,d**). Similarly, BC8 stained cells were dose-dependently depleted while CD45$^{I283M+H285R+N286D}$ base edited cells were shielded from all tested BC8-SAP concentrations (**Extended Data Fig. 3k,l**). Due to the complete non-binding of BC8 to CD45^{H285R} and CD45^{N286D} (**Fig. 1c,d**), the selective resistance of base edited cells was more apparent and equally resulted in an enrichment of correctly edited cells (**Extended Data Fig. 3m**). Thus, we identified base editable CD45 variants that shielded primary human T cells from cytotoxicity by a surrogate ADC. CD45^{K352E} displayed the most desirable characteristics for a genetically encoded shield, i.e. complete loss of binding to a very high affinity mAb (MIRG451) with preserved protein stability (**Fig. 1c-e**, **Extended Data Fig. 2b,c**). However, the editing efficiency of ABE8e-NG + sgRNA-49 (CD45$^{N351D+K352E}$) was very low.

1 Palchaudhuri, R. *et al.* Non-genotoxic conditioning for hematopoietic stem cell transplantation using a hematopoietic-cell-specific internalizing immunotoxin. *Nat Biotechnol* **34**, 738-745 (2016). https://doi.org:10.1038/nbt.3584

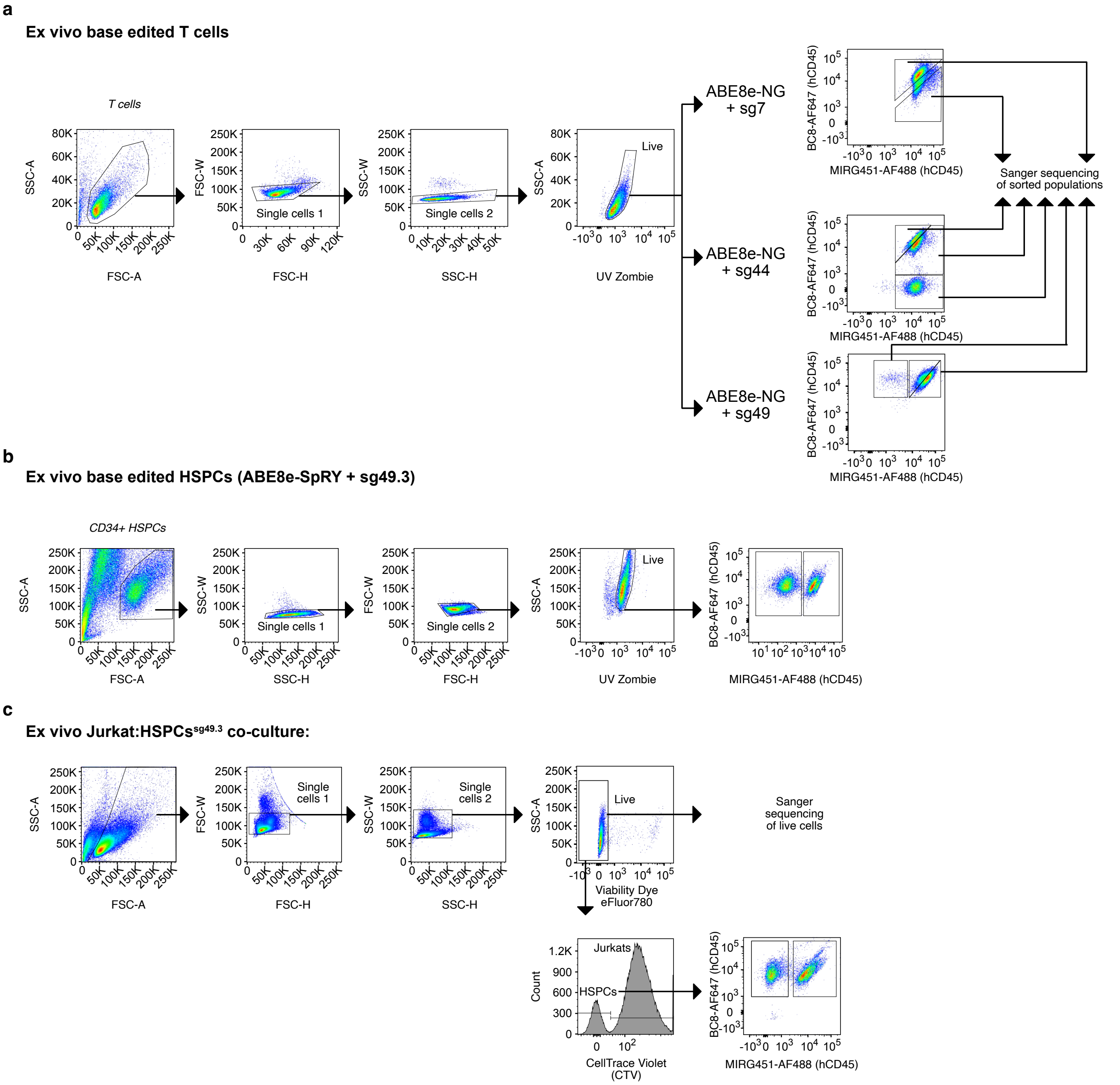

Supplementary Methods 1:

Flow cytometry gating strategy of ex vivo base edited human T cells (**a**) and HSPCs alone (**b**) or co-cultured with Jurkat cells (**c**). Sorted populations used for Sanger sequencing are depicted (**a, c**). Related to Figure 2, Extended Data Figure 3, 4 and 5.

a

Bone Marrow

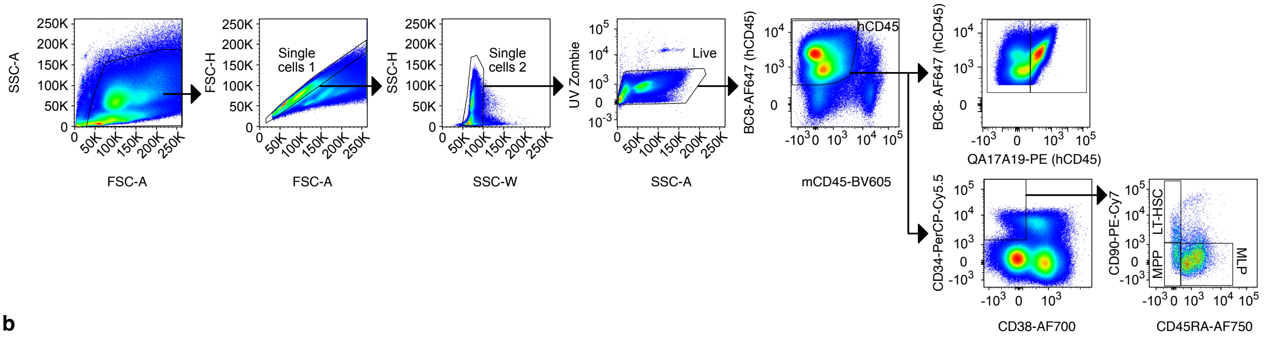

b

Spleen

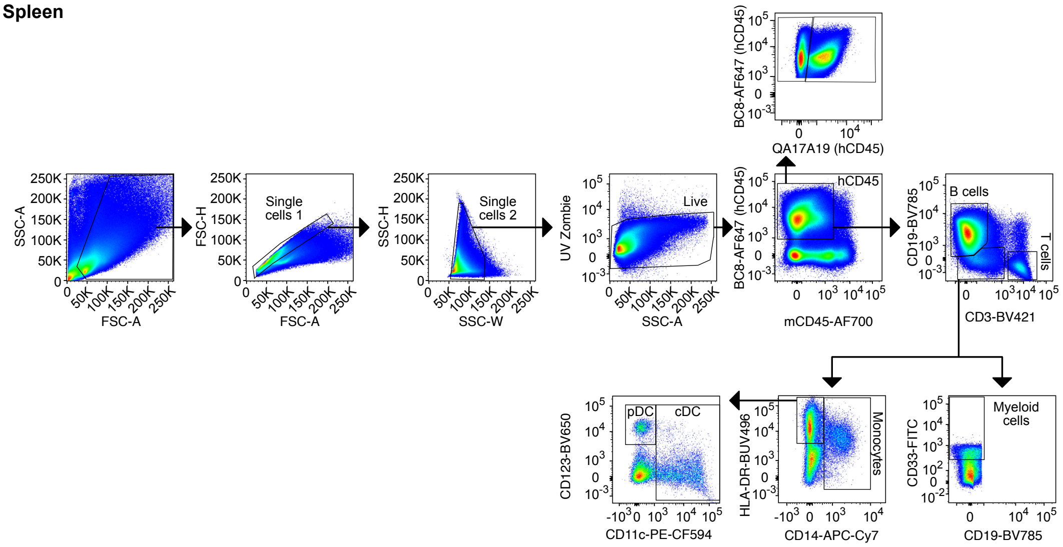

c

Blood

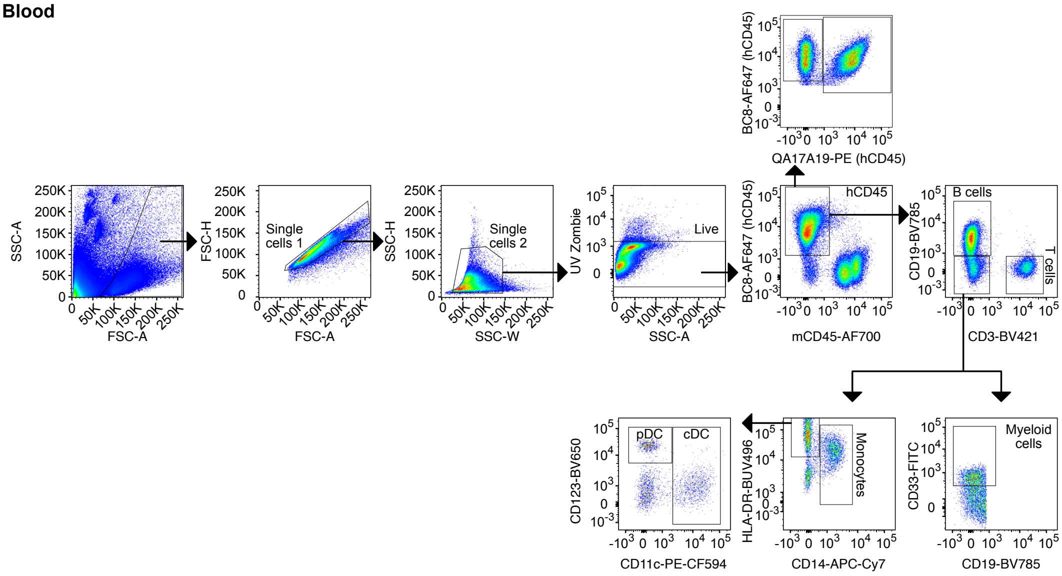

Supplementary Methods 2:

Flow cytometry gating strategy of bone marrow (**a**), spleen (**b**) and blood (**c**) from mice which received $^{sg49.3}$HSPCs and were treated with saline. Related to Figure 3 and Extended Data Figure 6.

a

Bone Marrow

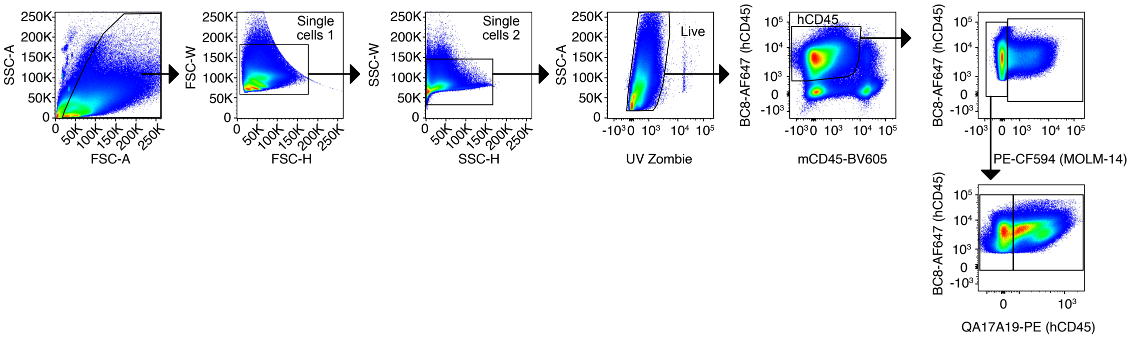

b

Spleen

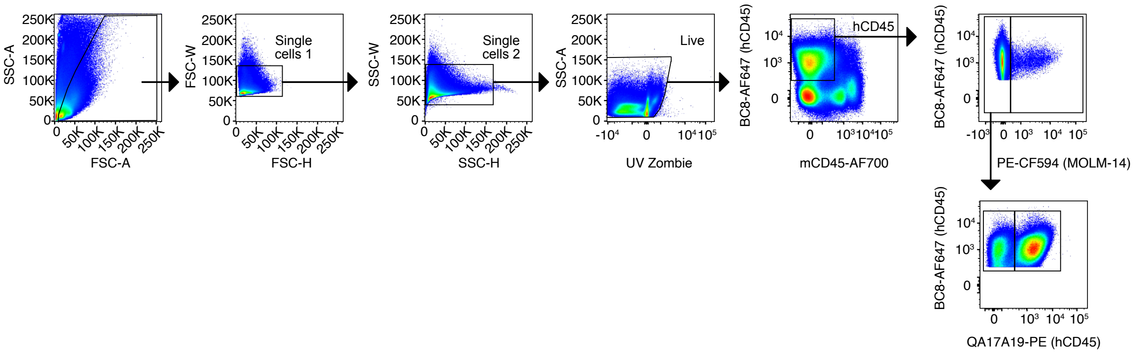

c

Blood

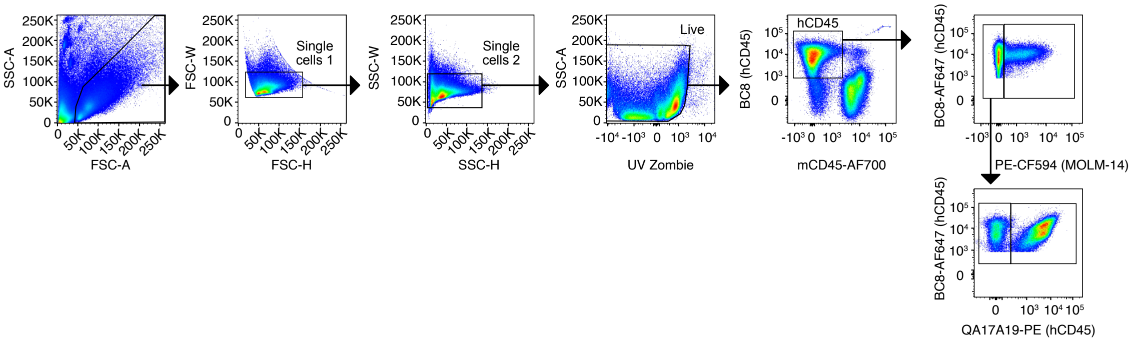

Supplementary Methods 3:

Flow cytometry gating strategy of bone marrow (**a**), spleen (**b**) and blood (**c**) from mice which received $^{sg49.3}$HSPCs, MOLM-14 and were treated with saline. Related to Figure 4, Extended Data Figure 7 and 8.

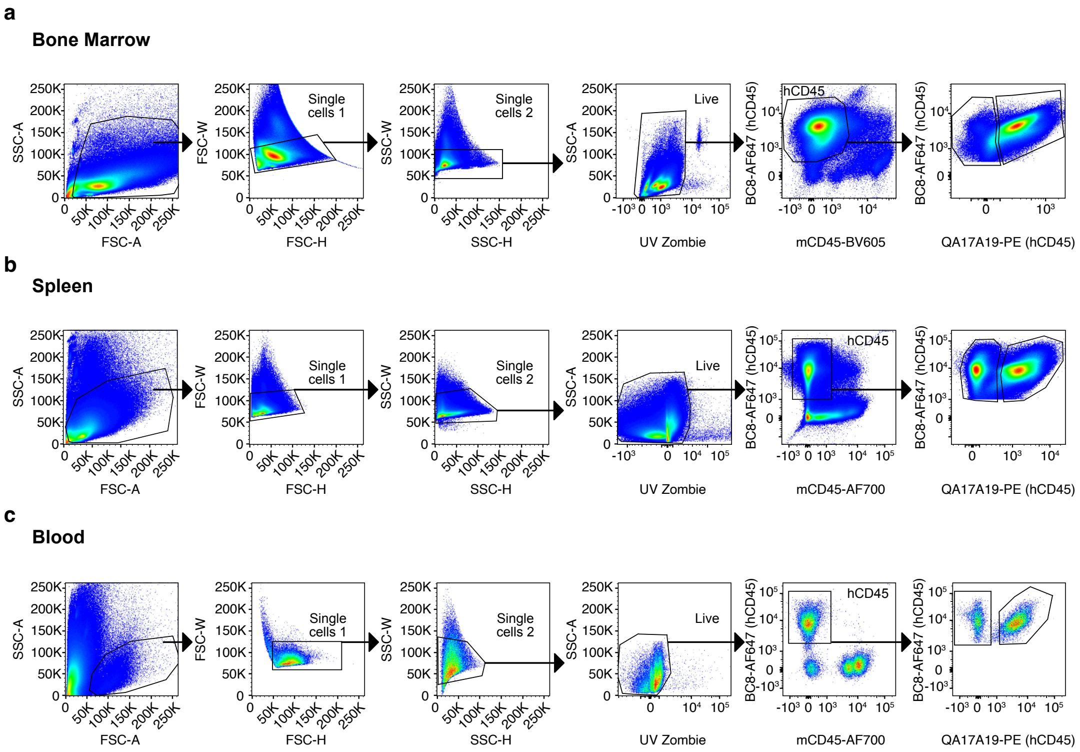

Supplementary Methods 4:

Flow cytometry gating strategy of bone marrow (**a**), spleen (**b**) and blood (**c**) from mice which received $^{sg49.3}$HSPCs, PDX and were treated with saline. Related to Figure 5 and Extended Data Figure 10.
