# Supplementary material for: Selective haematological cancer eradication with preserved haematopoiesis
Source: Nature. 2024 May 22;630(8017):728–35. doi: 10.1038/s41586-024-07456-3 (PMC11186773; doi:10.1038/s41586-024-07456-3)
Supplement: Supplementary file 2 — Reporting Summary [file 41586_2024_7456_MOESM2_ESM.pdf]

nature portfolio

Corresponding author(s): Lukas Jeker

Last updated by author(s): Mar 20, 2024

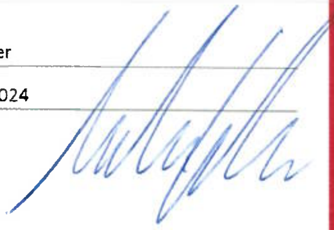

Reporting Summary

Nature Portfolio wishes to improve the reproducibility of the work that we publish. This form provides structure for consistency and transparency in reporting. For further information on Nature Portfolio policies, see our Editorial Policies and the Editorial Policy Checklist.

Statistics

For all statistical analyses, confirm that the following items are present in the figure legend, table legend, main text, or Methods section.

n/a	Confirmed	
☐	☒	The exact sample size (n) for each experimental group/condition, given as a discrete number and unit of measurement
☐	☒	A statement on whether measurements were taken from distinct samples or whether the same sample was measured repeatedly
☐	☒	The statistical test(s) used AND whether they are one- or two-sided<br>*Only common tests should be described solely by name; describe more complex techniques in the Methods section.*
☒	☐	A description of all covariates tested
☒	☐	A description of any assumptions or corrections, such as tests of normality and adjustment for multiple comparisons
☐	☒	A full description of the statistical parameters including central tendency (e.g. means) or other basic estimates (e.g. regression coefficient) AND variation (e.g. standard deviation) or associated estimates of uncertainty (e.g. confidence intervals)
☐	☒	For null hypothesis testing, the test statistic (e.g. F, t, r) with confidence intervals, effect sizes, degrees of freedom and P value noted<br>*Give P values as exact values whenever suitable.*
☒	☐	For Bayesian analysis, information on the choice of priors and Markov chain Monte Carlo settings
☒	☐	For hierarchical and complex designs, identification of the appropriate level for tests and full reporting of outcomes
☒	☐	Estimates of effect sizes (e.g. Cohen's d, Pearson's r), indicating how they were calculated

Our web collection on statistics for biologists contains articles on many of the points above.

Software and code

Policy information about availability of computer code

Data collection	Immunophenotypic analysis were performed on BD LSRFortessa using the BD FACS Diva software. Cell sorting was performed on a BD FACSAria or BD FACSMelody Cell Sorter; Antibody binding analysis were performed on a Octet system RED96e (Sartorius) or R8 (Sartorius); thermostability analysis were performed using Nanotemper Prometheus NT.48 NanoDSF or a Nanotemper Prometheus Panta (NanoTemper Technologies); Soluble proteins were purified using Äkta Pure (Cytiva) and monomer content was determined on a 1260 Infinity II HPLC system (Agilent); Luminescence measurements were performed on a BioTek Synergy H1 plate reader or for in vivo studies using a Newton7.0 imaging (Vilber); NGS libraries were sequenced on a MiniSeq (Illumina).
Data analysis	FlowJo 10.10.0; GraphPad Prism v10.1.1; Microsoft Office 365 Package; STEMvision Software Version 2021.05.07.00; Edit R v1.0.8; R v4.3.1; CRISPResso2 v2.2.14; R Studio v2023.06.2; ggplot2 v3.4.4; Kuant Vilber; binding data were analyzed using Octet Analysis Studio 12.2, thermal stability using PR. Panta analysis v1.7.4, monomer content using the Agilent 1280 Infinity II software or Cytiva Unicorn software; https://github.com/tsailabSJ/changeseq/tree/dev; https://github.com/tsailabSJ/MKSR_off_targets/blob/main/data/raw_data/crispressoPooled_allele_edit_eff.py; https://gitlab.com/JekerLab/cd45_shielding

For manuscripts utilizing custom algorithms or software that are central to the research but not yet described in published literature, software must be made available to editors and reviewers. We strongly encourage code deposition in a community repository (e.g. GitHub). See the Nature Portfolio guidelines for submitting code & software for further information.

Data

Policy information about availability of data

All manuscripts must include a data availability statement. This statement should provide the following information, where applicable:

- Accession codes, unique identifiers, or web links for publicly available datasets
- A description of any restrictions on data availability
- For clinical datasets or third party data, please ensure that the statement adheres to our policy

All data supporting the findings of this study are available within the paper and its Supplementary Information (including source data for main figures and Extended Data Figures reporting mouse data and FACS gating strategies) or public repositories. Datasets for targeted amplicon sequencing, CHANGE-SEQ-BE and rhAmp-Seq are available at the European Nucleotide Archive under the following accession number: PRJEB74081. The following public databases were used: ensembl human genome GRCh38.p14 (https://www.ensembl.org/Homo_sapiens/Info/Index) and protein sequence and structural data used in this study are publicly accessible through UniProtKB https://www.uniprot.org/uniprotkb/P08575/entry and PDB https://www.rcsb.org/structure/5FMV.

Research involving human participants, their data, or biological material

Policy information about studies with human participants or human data. See also policy information about sex, gender (identity/presentation), and sexual orientation and race, ethnicity and racism.

Reporting on sex and gender	Anonymous HSPCs donors were males. PDX sample was from a male patient.<br>The sex of PBMCs donor was not disclosed by the Blood Donation Center of the Basel University Hospital.
Reporting on race, ethnicity, or other socially relevant groupings	Please specify the socially constructed or socially relevant categorization variable(s) used in your manuscript and explain why they were used. Please note that such variables should not be used as proxies for other socially constructed/relevant variables (for example, race or ethnicity should not be used as a proxy for socioeconomic status).<br>Provide clear definitions of the relevant terms used, how they were provided (by the participants/respondents, the researchers, or third parties), and the method(s) used to classify people into the different categories (e.g. self-report, census or administrative data, social media data, etc.)<br>Please provide details about how you controlled for confounding variables in your analyses.
Population characteristics	For Extended Data Figure 9: There is a male predominance (74%), mean age of the patient cohort is 58.5 years. The patient population analysed includes 55% NPM1 positive patients.
Recruitment	For Extended Data Figure 9: Patients included in the MFI-analysis of CD45 in AML samples were included by retrospective screening of diagnostic bone marrow punctures for AML performed at the University Hospital Basel. Being a tertiary center performing induction chemotherapy and allogeneic HCT, there is bias for patients fit to undergo intensive chemotherapy which are referred from smaller centers. This does not affect the generalization of the data and represents the target population of potential clinical applications of our approach.
Ethics oversight	Anonymized HSPCs and PBMCs were purchased. For Figure 5 and Extended Data Figure 10: Deidentified patient-derived AML sample was obtained from the PDX repository (Cancer Research Center of Toulouse, France). A signed written informed consent for research use in accordance with the Declaration of Helsinki was obtained from patients and approved by the Geneva Health Department Ethic Committee. For Extended Data Figure 9: For AML patient samples all patients gave written informed consent to analysis of clinical data for research purposes, the study was approved by the local ethics committee (BASEC-Nr. 2023-01372).

Note that full information on the approval of the study protocol must also be provided in the manuscript.

Field-specific reporting

Please select the one below that is the best fit for your research. If you are not sure, read the appropriate sections before making your selection.

☒ Life sciences ☐ Behavioural & social sciences ☐ Ecological, evolutionary & environmental sciences

For a reference copy of the document with all sections, see nature.com/documents/nr-reporting-summary-flat.pdf

Life sciences study design

All studies must disclose on these points even when the disclosure is negative.

Sample size	No sample size calculations were performed to design the experiments. However, when possible, we aimed to have at least 5 replicates or mice per group to ensure sufficient data points for statistical analysis. For some in vivo experiments, the groups are smaller due to the limited number of available cells. For tumour experiments we initially used equal numbers of mice per group. Once the kinetics of a given tumour model was established in our setting we reduced the number of mice in the control groups to limit the number of mice with the most severe tumour burden (3R principles).
Data exclusions	Some data points of the in vivo experiments were excluded after visual inspection of samples if the FACS time gate showed irregularities. One mouse that did not engraft HSPCs was excluded from Fig. 5 and Extended Data Fig. 10. Cell numbers in the sgNTC group treated with CIM053-

SG3376 were so low that analysis of certain assays became unreliable (NGS, genetic chimerism analysis). We therefore excluded this group from NGS.

Replication — Number of biological replicates is specified for each experiment in the figure legend. Several key experiments were performed by different scientists, at times in different laboratories and reagents were shared. For instance, identification of variants, characterization of recombinant variants and FACS validation were performed by different scientists. Some experiments were performed in the academic lab and validated in Cimeio labs and vice-versa. Experiments were in general independently performed at least twice with a few exceptions (e.g. secondary transplants in vivo was performed once). All attempts to replicate the date were successful.

Randomization — To avoid unconscious bias assigning mice to saline or treated groups, we deliberately assigned mice with the largest tumor mass to the CIM053-SG3376 treated groups. For all other experiments not involving tumours, sample allocation was random.

Blinding — Investigators were not blinded during in vivo data acquisition/analysis as saline control groups had to be monitored for tumour growth and clinical score upon euthanasia. The investigator who determined genetic chimerism (NGS and analysis) was blinded and provided the results to the investigator in charge of supervising in vivo experiments. The investigators performing CHANGE-Seq_BE, rhAMPSeq and analysed the data were blinded and provided the results to the investigator in charge of supervising in vivo experiments.

Reporting for specific materials, systems and methods

We require information from authors about some types of materials, experimental systems and methods used in many studies. Here, indicate whether each material, system or method listed is relevant to your study. If you are not sure if a list item applies to your research, read the appropriate section before selecting a response.

Materials & experimental systems

n/a	Involved in the study	
☐	☒	Antibodies
☐	☒	Eukaryotic cell lines
☒	☐	Palaeontology and archaeology
☐	☒	Animals and other organisms
☒	☐	Clinical data
☒	☐	Dual use research of concern
☒	☐	Plants

Methods

n/a	Involved in the study	
☒	☐	ChIP-seq
☐	☒	Flow cytometry
☒	☐	MRI-based neuroimaging

Antibodies

Antibodies used — Antibodies and fluorochromes RRIDs can be found in Extended Data Table 6.

Validation — All commercial antibodies were validated by the manufacturing companies (Biolegend, BD Biosciences, Beckman Coulter, Ichor Bio, Invitrogen, Miltenyi, BioXcell). Each product comes with a certificate of analysis from the indicated vendor stating that the product has met all quality controls standards. Antibodies used for the analysis of in vivo experiments, FMO controls were included to confirm specific binding. MIRG451 and CIM053 were validated on CRISPR CD45 knock-out Jurkat cells by FACS where they did not show any staining. MIRG451 and CIM053 were also validated on recombinant human CD45; no cross-reactivity with mouse CD45 was detected.

Eukaryotic cell lines

Policy information about cell lines and Sex and Gender in Research

Cell line source(s) — Cell lines were purchased from ATCC or DSMZ and RRIDs can be found in Extended Data Table 5.

Authentication — All cell lines were STR profiled or purchased from ATCC or DSMZ.

Mycoplasma contamination — All the cell lines used were tested mycoplasma negative.

Commonly misidentified lines (See ICLAC register) — No commonly misidentified cell lines were used.

Animals and other research organisms

Policy information about studies involving animals; ARRIVE guidelines recommended for reporting animal research, and Sex and Gender in Research

Laboratory animals — NSG-SGM3 (stock #013062) and NBSGW (stock #026622) mice were purchased form Jackson Laboratories. Mice were 4-6 weeks at the start of the experiments (ages are indicated in each figure). Mice were kept on artificial light with daylight spectra, 12 hours light/12 hours dark with dusk and dawn phases of 30 minutes, maximum 200 lux. The temperature is between 20°C and 24°C and the humidity is 45-65%. Experiments were performed during the light phase.

Wild animals	No wild animals were used in the study.
Reporting on sex	Female mice were used for in vivo studies.
Field-collected samples	No field collected samples were used in the study.
Ethics oversight	All animal work was performed in accordance with the federal and cantonal laws of Switzerland. Protocols were approved by the Animal Research Commission of the Canton of Basel-Stadt, Switzerland.

Note that full information on the approval of the study protocol must also be provided in the manuscript.

Flow Cytometry

Plots

Confirm that:

☒ The axis labels state the marker and fluorochrome used (e.g. CD4-FITC).

☒ The axis scales are clearly visible. Include numbers along axes only for bottom left plot of group (a 'group' is an analysis of identical markers).

☒ All plots are contour plots with outliers or pseudocolor plots.

☒ A numerical value for number of cells or percentage (with statistics) is provided.

Methodology

Sample preparation	Flow cytometry analysis were recorded using BD LSRFortessa instruments with the BD FACSDiva software. Recorded files were analyzed with the FlowJo software. Antibodies used for flow cytometry are listed in the Extended Data Table 5. Cells were sorted with BD FACSAria III or BD FACSMelody Cell Sorter instruments. Cells were first collected and washed in PBS then stained for viability 15 min in the dark; washed with PBS and then incubated with the adequate staining mix for 20 min in the dark at room temperature. Cells were then washed again with PBS and resuspended in 100 uL FACS buffer (PBS; 2% heat-inactivated FCS; 0.02% NaN3)
Instrument	BD LSRFortessa Cell Analyzer; BD FACSAria III Cell Sorter; BD FACSMelody Cell Sorter
Software	FlowJo Software (BD Life Sciences)
Cell population abundance	No purity check was performed after FACS cells sorting due to the low number of cells but sequencing results confirmed that the sorted populations were highly pure.
Gating strategy	Gating strategy for the main populations and sorted populations are shown in Supplementary methods 1-4.

☒ Tick this box to confirm that a figure exemplifying the gating strategy is provided in the Supplementary Information.
